# A murine MHC-Ib molecule, H2-Q9, drives peptide-centric T-cell receptor recognition of a viral antigen

**DOI:** 10.1016/j.jbc.2026.113574

**Published:** 2026-09-17

**Authors:** Luke Tennant, Chacko Jobichen, Mai T. Tran, Carine Farenc, Stephanie Gras, Aron E. Lukacher, Lucy C. Sullivan, Andrew G. Brooks, Jamie Rossjohn

**Affiliations:** 1 Infection and Immunity Program & Department of Biochemistry and Molecular Biology, Monash Biomedicine Discovery Institute, Monash University, Clayton, Victoria, Australia; 2 Department of Cell and Biological Systems, Penn State College of Medicine, Hershey, Pennsylvania, USA; 3 Department of Microbiology and Immunology, University of Melbourne, Parkville, Victoria, Australia

**Keywords:** major histocompatibility complex (MHC), antigen presentation, T-cell receptor (TCR), X-ray crystallography, crystal structure, peptide recognition, nonclassical MHC class Ib, H2-Q9 (Qa-2), mouse polyomavirus (MuPyV), CD8+ T cell

## Abstract

Highly polymorphic classical major histocompatibility complex class I (MHC-Ia) molecules present peptides to T-cell receptors (TCRs) expressed on CD8^+^ T-cells. In contrast, nonclassical MHC-Ib molecules are less polymorphic, and the structural principles governing their recognition by TCRs remain poorly understood. H2-Q9, a murine Qa-2 family MHC-Ib molecule presents the mouse polyomavirus-derived VP2.139 peptide (HALNVVHDW) to CD8^+^ T-cells, yet the molecular basis of TCR recognition of H2-Q9/peptide complexes remains unclear. Here, we characterized the interactions of two VP2.139-specific TCRs, C3K and AH1, with the VP2.139 peptide presented by H2-Q9, which showed similar moderate binding affinities. We then determined the structure of the TCR C3K bound to the VP2.139 peptide presented by H2-Q9. TCR C3K adopted a diagonal docking orientation and was tilted toward the N-terminal end of the peptide. The CDR3 loops formed extensive contacts with peptide positions P1–P8, whereas alanine-scanning mutagenesis at positions P1–P7 impaired TCR recognition. Structural comparison of H2-Q9 with classical murine MHC-Ia molecules identified substitutions in the α3-domain that alter the CD8-binding interface and may explain the previously observed absence of CD8 binding to H2-Q9. Together, these findings provide insight into antigen recognition by a monomorphic MHC-Ib molecule.

Major histocompatibility complex class I (MHC-I) molecules present intracellularly derived peptides on the cell surface for surveillance by CD8^+^ T-cells (1). MHC-I molecules are typically divided into two classes, classical MHC-Ia and nonclassical MHC-Ib. Classical MHC-Ia including HLA-A, HLA-B, and HLA-C in humans and H2-K, H2-D, and H2-L in mice, are highly polymorphic, broadly expressed, and specialized for the presentation of diverse pathogen- and self-derived peptides. In contrast, MHC-Ib molecules (HLA-E, -F, -G, and -H in humans and H2-M, -Q, and -T in mice) generally exhibit more limited polymorphism, more restricted expression patterns, and specialized immunological functions (2). These specialized functions included presenting constrained peptide or nonpeptide ligands, regulating natural killer and T-cell responses, and modulating immune activity in specific developmental or tissue contexts. Thus, although MHC-Ib molecules share the overall MHC-Ia fold, they provide an opportunity to understand how antigen presentation and T-cell recognition can be adapted beyond the canonical classical MHC-Ia paradigm.

The murine *H2-Q* region encodes several nonclassical MHC-Ib molecules, including members of the Qa-2 family. H2-Q9, hereafter Q9, is a Qa-2 family molecule that is attached to the cell surface *via* a glycosylphosphatidylinositol anchor rather than a conventional transmembrane domain (3). Qa-2 molecules have been proposed to serve as a functional homologue of human HLA-G, with both systems implicated in immune regulation and embryonic development rather than in broad classical antigen presentation (4). This functional relationship is supported by shared biological features, including MHC-Ib identity, restricted polymorphism, membrane-bound and soluble forms, and roles in reproductive or immunoregulatory contexts (5, 6, 7, 8). Q9 is also closely related to classical murine MHC-Ia molecules at the sequence level, with reported identities of 84%, 82%, and 82% to H2-D^b^, H2-K^b^, and H2-L^d^, respectively (9), emphasizing that Q9 combines a classical MHC-I-like scaffold with nonclassical biological function.

Unlike many nonclassical MHC-Ib molecules, which present limited ligand repertoires, Q9 can accommodate a relatively broad set of peptides (10). Structural studies of Q9 suggested that this capacity is enabled by features of the peptide-binding groove that permit diverse ligands whereas maintaining peptide conformations suitable for immune recognition (11). Among these ligands, Q9 presents the dominant polyomavirus-derived peptide VP2.139 to CD8^+^ T-cells (2). The closely related molecule Q7 has been reported to support T-cell responses with reduced dependence on CD8 coreceptor engagement compared with classical MHC-Ia-restricted responses (12), suggesting that Q9-restricted responses may exhibit similar properties, given the 99% sequence identity between Q7 and Q9. Furthermore, Q9-restricted CD8^+^ T-cells exhibited atypical functional properties, including limited interferon-γ production after antigen stimulation (13). These findings and observations from closely related Qa-2 molecules suggested that Q9 may shape T-cell activation through mechanisms distinct from those used by classical MHC-Ia molecules.

In addition to their atypical functional properties, Q9-restricted T-cell responses exhibited a distinctive T-cell receptor (TCR) repertoire. Previous studies have shown that Q9-restricted TCRs utilize a diverse range of Vα gene segments, whereas Vβ is markedly biased toward *TRBV13* and *TRBV29* (14). This restricted β-chain usage suggested that recognition of Q9/peptide complexes may impose unique structural constraints on TCR engagement. However, the molecular basis for this apparent repertoire bias and its relationship to antigen recognition remains poorly understood. More broadly, the mechanisms underlying the atypical functional properties of Q9-restricted T-cells remains unresolved. Several mechanisms could explain these unusual Q9-restricted responses, including altered thymic selection leading to reduced effector function, impaired CD8 engagement arising from structural differences in Q9, or distinct TCR docking modes that alter the signaling outcomes of antigen recognition (15, 16). The physiological relevance of one such Q9-restricted receptor, TCR C3K, has been demonstrated *in vivo* using retrogenic mice, in which TCR C3K-expressing CD8^+^ T-cells expanded following infection with WT murine polyomavirus (MuPyV) but not with a virus carrying an anchor mutation in the VP2.139 epitope (17). These findings establish TCR C3K recognition of Q9/VP2.139 as a physiologically relevant antiviral interaction and provide a basis for investigating its molecular determinants.

To begin to address some of these fundamental questions, we characterize two TCRs exhibiting distinct gene usage, C3K and AH1, which showed similar binding affinities. We also provide insight into the structural basis of TCR recognition of the MuPyV VP2.139 peptide bound to Q9. Using alanine-scanning mutagenesis of the VP2.139 peptide and crystal structure analyses, we found that residues P1 to P7 (except P2) are critical for both the TCRs C3K and AH1 responses. In addition, a structural analysis of Q9/VP2.139 and H2-K^b^ in complex with CD8αα revealed a distinct surface interface on the α3-domain for CD8αα binding between Q9 and H2-K^b^. These differences may explain how Q9 can support antigen-specific T-cell recognition of a conventionally processed viral peptide whereas diverging from canonical CD8-dependent classical MHC-Ia recognition.

## Results

### Q9-restricted TCRs bind Q9/VP2.139 with moderate micromolar affinity

To begin understanding the molecular basis of Q9-restricted TCR recognition, we first expressed and purified the extracellular domains of the MuPyV-specific TCRs C3K and AH1, together with Q9 refolded with the viral peptide VP2.139. The TCR sequences of C3K and AH1 were obtained from a previous study in which they were isolated from CD8^+^ T-cell hybridomas (17). TCR C3K utilizes *TRAV13D-1∗04* and *TRBV2∗01* gene segments, whereas TCR AH1 utilizes *TRAV14-2∗02* and *TRBV13-3∗01* (Table S1).

We then used surface plasmon resonance (SPR) to determine whether Q9-restricted TCRs bind Q9/VP2.139 with affinities typical of TCR–peptide–MHC-Ia (pMHC-Ia) interactions (1). Despite different kinetic profiles, TCRs C3K and AH1 bound Q9/VP2.139 with similar, moderate micromolar affinities, with equilibrium dissociation constants of 41.1 ± 3.8 μM for TCR C3K and 32.3 ± 3.5 μM for TCR AH1 (Fig 1 *A–D*, Table 1 and Figs S1–S4). These affinities fall within the typical physiological range reported for TCR–pMHC-Ia interactions (1), indicating that both receptors engage Q9/VP2.139 with affinities suitable for productive TCR recognition.

**Figure 1:**
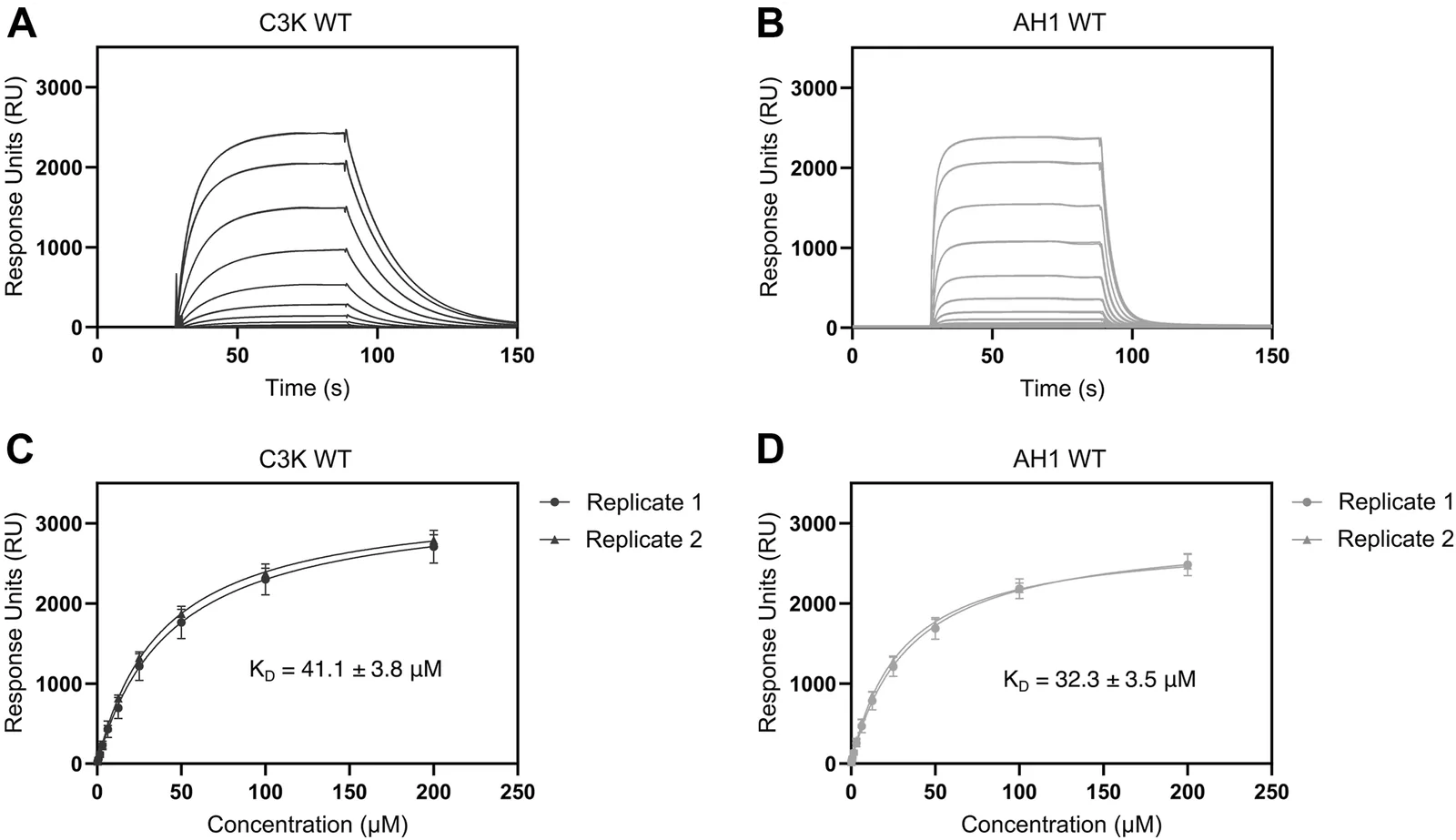
**Binding affinities of Q9-restricted TCRs.** Measured response curves for single dilution series of TCR C3K (*A*) and TCR AH1 (*B*) to WT Q9/VP2.139 were determined using SPR. H2-K^b^ presenting the pBM1 peptide (amino acid sequence: INFDFNTI) was included as a negative control. Curve fits for TCR C3K–Q9/VP2.139 (*C*) and TCR AH1–Q9/VP2.139 (*D*) and K_D_ determination using a 1:1 steady-state binding model, R_eq_ = R_max_[A]/K_D_+[A], where R_max_ is the maximum binding response, [A] is the analyte concentration, and K_D_ is the equilibrium dissociation constant. Replicates 1 and 2 represent two independent SPR experiments. Within each independent experiment, each TCR sample was analyzed in duplicate. Data are presented as mean ± standard error of the mean (SEM) from the two independent experiments (n = 2). SPR, surface plasmon resonance; TCR, T-cell receptor.

**Table 1:** Q9-restricted TCR binding affinities for Q9/VP2.139 and alanine mutants at positions P1–P8 of the peptide

Mutant	C3K affinity (K_D_)	ΔΔG_eq_ (kcal/mol)	Effect on affinity	AH1 affinity (K_D_)	ΔΔG_eq_ (kcal/mol)	Effect on affinity
WT	41.1 ± 3.8 μM			32.3 ± 3.5 μM		
H1A	NB	>>0.94	c	NB	>>1.08	c
L3A	NB	>>0.94	c	139.2 ± 8.1 μM	0.87	c
N4A	NB	>>0.94	c	NB	>>1.08	c
V5A	NB	>>0.94	c	167.2 ± 9.7 μM	0.97	c
V6A	187.1 ± 0.5 μM	0.90	c	NB	>>1.08	c
H7A	NB	>>0.94	c	NB	>>1.08	c
D8A	55.7 ± 0.3 μM	0.18	a	38.2 ± 1.9 μM	0.10	a

NB, nonbinding; TCR, T-cell receptor. Mutants with K_D_ >200 μM (outside the reliable detection range) or with no detectable binding response were classified as nonbinding. ΔΔG_eq_ = R × T × ln(K_D mutant_/K_D WT_), where R is the gas constant and T is the temperature in Kelvin. Effect on binding affinity is considered either minimal,^a^ moderate,^b^ or strong.^c^

a More than 50% of the WT value, minimal.

b Between 50% and 20% of the WT value, moderate.

c Less than 20% of the WT value, strong.

**Figure 2:**
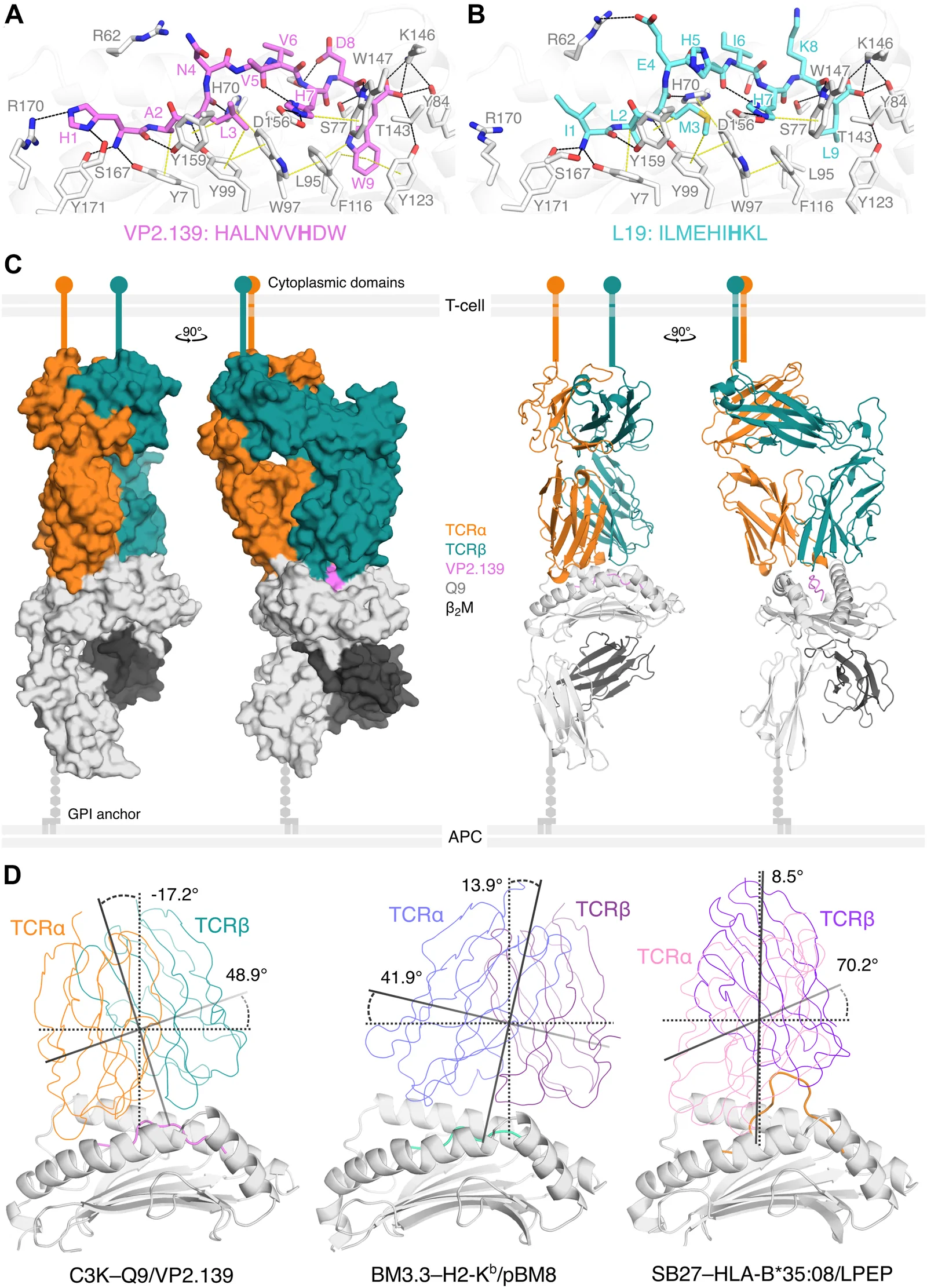
**Structural basis of VP2.139 presentation and recognition by TCR C3K.** Q9 presented the (*A*) VP2.139 viral peptide (PDB: 43RZ) in an orientation similar to that of the (*B*) L19 self-peptide (PDB: 1K8D). Hydrogen bonds and π-interactions are indicated by *black* and *yellowdashed lines*, respectively. (*C*) Surface (*left*) and cartoon (*right*) representation of the C3K–Q9/VP2.139 complex (PDB: 43RH). The TCR C3K α-chain and β-chain are shown in *orange* and *dark teal*, respectively. The Q9 MHC heavy-chain, β_2_M, and the VP2.139 peptide are colored in *light gray*, *dark gray*, and *pink*, respectively. (*D*) Comparison of the docking geometries of TCR C3K over Q9/VP2.139 (*left*), TCR BM3.3 over H2-K^b^/pBM8 self-peptide (*middle*, PDB: 2OL3), and TCR SB27 over HLA-B∗35:08/LPEP viral peptide (*right*, PDB: 2AK4). *Solid orthogonallines* indicate the TCR interdomain axes of the variable subunits, and *dotted orthogonal lines* indicate the pMHC groove and normal vectors. Q9, H2-K^b^, and HLA-B∗35:08 are colored *gray*. MHC, major histocompatibility complex; PDB, Protein Data Bank; TCR, T-cell receptor.

**Figure 3:**
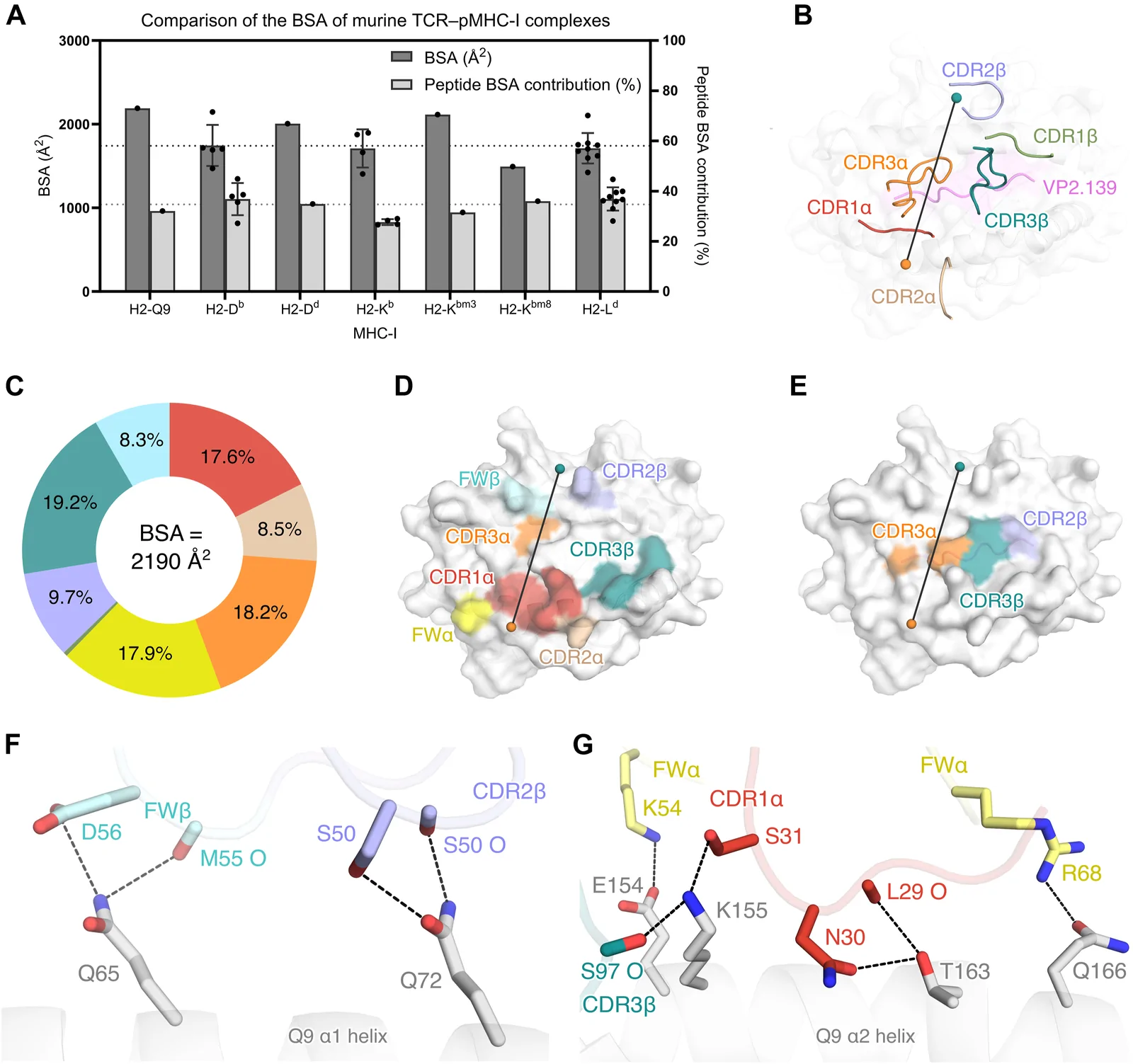
**TCR C3K interaction footprint on Q9/VP2.139 and the interactions with Q9.** (*A*) Comparison of the total BSA (*dark gray*) and the percentage peptide contribution (*light gray*) among murine TCR–pMHC-I complexes, including the C3K–Q9/VP2.139 complex. (*B*) Surface view of the antigen-presenting platform showing the binding interface and the docking orientation of TCR C3K over Q9/VP2.139. Throughout, the CDR loops, 1α, 2α, 3α, 1β, 2β, and 3β are colored in *vermillion*, *wheat*, *orange*, *green*, *light blue*, and *dark teal*, respectively. Framework (FW) α and β regions are colored *light yellow* and *light cyan*, respectively. Q9 and VP2.139 are colored *light gray* and *pink*, respectively. Centers of mass of the TCR C3K Vα- and Vβ-domains are indicated by *spheres*, with a connecting line illustrating the crossing angle. (*C*) The pie chart presents the relative BSA contribution of each CDR loop and FW region of the TCR C3K to the Q9/VP2.139 interface. Atomic footprint analysis of CDR loop contacts with the (*D*) Q9 MHC heavy-chain and (*E*) VP2.139 peptide across the interface. TCR-proximal regions of the pMHC surface are colored according to the contacting CDR loops. Close-up views of the C3K–Q9/VP2.139 interface for the Q9 (*F*) α1 and (*G*) α2 helices. Key side-chain interactions between Q9 and the TCR CDR loops are shown as *sticks*, with hydrogen bonds indicated as *blackdashedlines*. BSA, buried surface area; CDR, complementarity-determining region; MHC, major histocompatibility complex; TCR, T-cell receptor.

**Figure 4:**
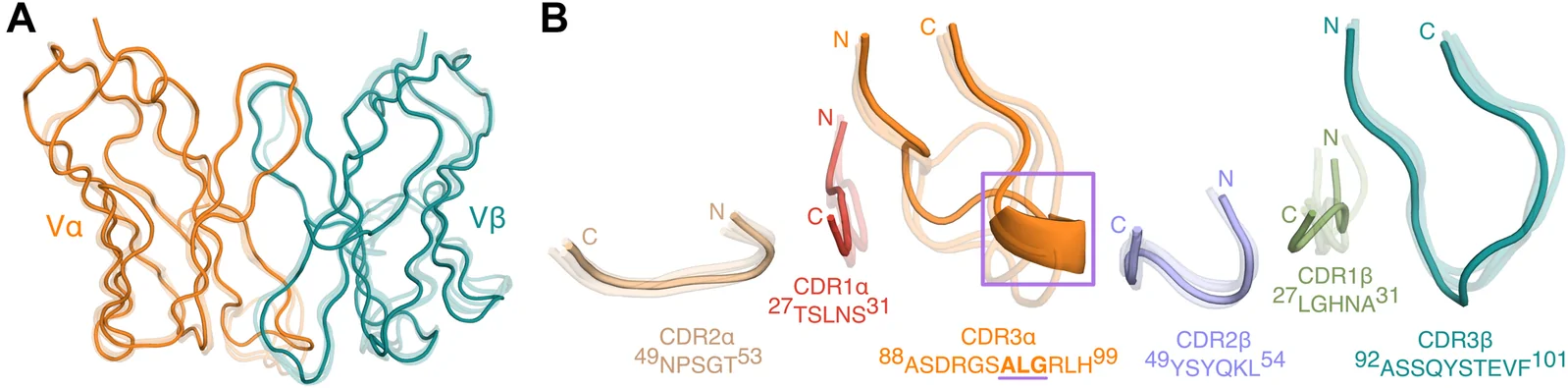
**TCR C3K engagement induces CDR3α remodeling and reorganization of the Q9/VP2.139 interface.** Structural superposition of the (*A*) variable domains and (*B*) CDR loops of the TCR C3K bound (*opaque*) and unbound (*semitransparent*) to Q9/VP2.139. The variable domains and CDR loops are shown as ribbon diagrams. The CDR loops, 1α, 2α, 3α, 1β, 2β, and 3β are colored in *vermillion*, *wheat*, *orange*, *green*, *light blue*, and *dark teal*, respectively. *Purple* box denotes the corresponding regions of the CDR3α loop that forms a 3_10_-helix. CDR, complementarity-determining region; TCR, T-cell receptor.

### Q9 presents VP2.139 in a pronounced bulged conformation

To define the positioning of VP2.139 in the peptide-binding groove of Q9 in the absence of TCR, the crystal structure of Q9/VP2.139 was determined at 2.00 Å resolution (Figs. 2*A*, S5, and Table 2). The binary structure revealed that VP2.139 adopts a pronounced bulged conformation within the Q9 peptide-binding groove, with several residues anchored within the Q9 groove and others exposed for potential TCR contact (Fig. 2*A*). Specifically, P2-Ala, P3-Leu, P7-His, and P9-Trp were buried within the Q9 binding groove, whereas P1-His, P4-Asn, P5-Val, P6-Val, and P8-Asp were solvent exposed and therefore accessible for potential TCR contact (Fig. 2*A*).

**Table 2:** X-ray diffraction data collection and refinement statistics of C3K–Q9/VP2.139 complex and binaries

	C3K–Q9/VP2.139	C3K	Q9/VP2.139	Q9/VP2.139 P1A	Q9/VP2.139 P4A	Q9/VP2.139 P5A	Q9/VP2.139 P8A
Data collection							
Space group	C 1 2 1	P 1 21 1	P 21 21 21	P 21 21 21	P 21 21 21	P 21 21 21	P 21 21 21
Cell dimensions							
a, b, c (Å)	275.695, 66.810, 90.596	63.834, 102.889, 76.869	54.714, 57.288, 120.909	54.619, 56.799, 119.314	54.489, 56.827, 119.52	54.594, 56.99, 119.968	54.253, 56.534, 118.722
α, β, γ (°)	90, 98.84, 90	90, 106.47, 90	90, 90, 90	90, 90, 90	90, 90, 90	90, 90, 90	90, 90, 90
Resolution range (Å)	29.99–3.33 (3.6–3.33)	41.65–2.01 (2.06–2.01)	40.57–2.00 (2.01–2.00)	41.14–2.03 (2.08–2.03)	41.18–2.03 (2.08–2.03)	41.32–1.80 (1.84–1.80)	40.94–2.29 (2.37–2.29)
Rp.i.m (%)	8.2 (37.3)	2.4 (15.5)	2.6 (8.6)	2.4 (30.2)	1.8 (16.7)	1.5 (30.7)	2.5 (29.5)
Mn(I) half-set correlation CC1/2	0.991 (0.771)	0.999 (0.942)	0.999 (0.990)	0.999 (0.754)	1.000 (0.908)	1.000 (0.816)	0.999 (0.818)
I/σI	8.0 (2.3)	12.2 (2.9)	29.1 (11.6)	16.0 (2.4)	24.0 (4.5)	29.4 (2.7)	24.4 (2.8)
Total observations	104209 (20935)	90151 (5940)	366866 (28565)	179675 (12803)	177181 (12457)	255615 (14803)	122656 (11748)
Unique reflections	23857 (4850)	50006 (3566)	26306 (1937)	24708 (1766)	24808 (1757)	35337 (2071)	17074 (1639)
Completeness (%)	98.9 (99.0)	78.7 (76.4)	99.5 (100)	99.9 (99.2)	99.8 (98.5)	99.8 (99.4)	99.8 (98.4)
Multiplicity	4.4 (4.3)	1.8 (1.7)	13.9 (14.7)	7.3 (7.2)	7.1 (7.1)	7.2 (7.1)	7.2 (7.2)
Refinement							
Rwork (%)	21.8	18.7	21.2	20.9	20.6	18.7	21.9
Rfree (%)	26.7	22.4	24.1	24.6	24.1	23.0	25.2
Biological complexes in ASU	1	2	1	1	1	1	1
Number of atoms							
Protein	10,125	6970	3149	3132	3162	3147	3157
Water	0	525	218	38	50	76	20
Bond lengths (Å)	0.01	0.01	0.01	0.01	0.01	0.01	0.01
Bond angles (°)	0.85	0.95	1.06	1.10	1.05	0.90	1.24
Ramachandran plot							
Favored (%)	91.93	98.05	97.09	96.30	97.62	97.35	98.41
Allowed (%)	7.75	1.84	2.91	3.70	2.38	2.65	1.59
Outlier (%)	0.32	0.11	0.00	0.0	0.0	0.00	0.0

R_p.i.m_ = ∑_hkl_ [1/(N-1)]^1/2^ ∑_i_ | I_hkl_, _i_-<I_hkl_> |/∑_hkl_ <I_hkl_>. R_factor_ = (∑ | |F_o_| - |F_c_| |)/(∑ |F_o_|). Ten percent of data were used for the R_free_ calculation. Values in parentheses refer to the highest resolution bin.

Despite its distinct primary sequence, VP2.139 adopted an overall architecture closely resembling that of the previously characterized Q9-bound self-peptide L19 in its overall mode of presentation (Fig. 2*B*) (11). Both peptides adopted similar bulged conformations and formed analogous peptide–MHC contacts that stabilized their positioning within the Q9 groove (Fig 2, *A* and *B*).

We next compared Q9-bound peptides with peptides presented by other murine MHC-I molecules (Fig. S6). The peptide main-chain at P4 protruded substantially farther from the peptide-binding cleft in Q9 than in the other murine MHC-I structures examined, quantitatively supporting the pronounced central bulge observed for both VP2.139 and L19 (Fig. S6).

To determine whether this mode of peptide presentation was associated with distinctive features of the Q9 peptide-binding platform, we compared peptide-binding groove volumes across available murine MHC-I structures (Table S2). Q9 exhibited the largest calculated groove volume among the molecules examined (810.6 ± 50.1 Å^3^), compared with H2-Q10 (560.0 ± 40.6 Å^3^), H2-D^b^ (580.0 ± 50.4 Å^3^), H2-D^d^ (690.8 ± 40.2 Å^3^), H2-K^b^ (710.7 ± 20.4 Å^3^), H2-K^d^ (520.0 ± 30.7 Å^3^), H2-K^k^ (680.0 ± 20.9 Å^3^), and H2-L^d^ (650.3 ± 80.3 Å^3^) (Table S2).

The larger calculated volume of the Q9 groove did not reflect increased groove depth. Rather, although the Q9 peptide-binding groove was relatively shallow compared with that of H2-K^b^, it extended farther longitudinally at both the N- and C-terminal ends (Fig. S7).

Together, these findings indicate that Q9 possesses a relatively shallow but longitudinally extended peptide-binding groove that accommodates a pronounced bulged mode of peptide presentation.

### Q9-restricted TCR C3K adopts a canonical diagonal docking mode with an unusual N-terminal tilt

To define how a Q9-restricted TCR engages Q9/VP2.139, we determined the crystal structure of the C3K–Q9/VP2.139 complex at 3.33 Å resolution (Fig. 2*C*, Table 2, and Fig. S8). TCR C3K docked over Q9/VP2.139 with a crossing angle of 48.9°, consistent with the canonical docking polarity observed for most MHC-Ia-restricted TCRs (Fig. 2*D* and Table S3).

In contrast to its conventional crossing angle, C3K exhibited an incidence angle of −17.2° and was the only TCR among the published murine TCR–pMHC-Ia complexes surveyed to exhibit an N-terminal tilt (Table S3). The remaining murine TCRs tilted toward the C-terminal half of their bound peptides, including BM3.3–H2-K^b^/pBM8, which exhibited a C-terminal incidence angle of 13.9° (Fig. 2*D*). For additional comparison, we included the human-derived SB27–HLA-B∗35:08/LPEP complex, which similarly exhibited a C-terminal tilt, with an incidence angle of 8.5° (Fig. S2*D*). The opposing orientation of TCR C3K therefore corresponded to angular differences of 31.1° relative to BM3.3 and 25.7° relative to SB27.

The N-terminal tilt positioned TCR C3K centrally over the most prominently exposed region of the bulged VP2.139 peptide. Thus, although TCR C3K retains the diagonal docking polarity characteristic of conventional TCR–pMHC-Ia recognition, it adopts an unusual incidence angle that aligns the TCR over the pronounced central bulge of Q9-bound VP2.139.

### TCR C3K forms an extensive interface with Q9/VP2.139

TCR C3K recognized Q9/VP2.139 with an average buried surface area (BSA) of 2190 Å^2^, among the largest reported interfaces for murine class I-restricted TCR–pMHC complexes (Fig. 3*A* and Table S3). The peptide contributed approximately 32.1% of the total BSA, placing it within the expected range for murine TCR–pMHC-Ia structures (Fig. 3*A* and Table S3). The TCR α-chain was dominant in the interaction, contributing 56.9% of the BSA, whereas the TCR β-chain contribution was 43.1%.

All complementarity-determining region (CDR) loops except CDR1β contacted Q9/VP2.139 (Fig 3, *B–E*). The VP2.139 peptide was extensively buried within the center of the interface, with both CDR3 loops positioned over its exposed surface (Fig. 3*E*).

### TCR C3K contacts both Q9 α helices

TCR C3K contacted both the α1 and α2 helices of Q9 through residues in its framework and CDR regions. On the α1 helix, the side-chain of Q9-Gln65 was positioned within hydrogen bonding distance of the TCRβ-Asp56 side-chain and the main-chain carbonyl of TCRβ-Met55, consistent with a potential hydrogen bonding network (Fig. 3*F* and Table S4). Q9-Gln72 was also positioned near CDR2β, with its side-chain within hydrogen bonding distance of both the side-chain hydroxyl and main-chain carbonyl of TCRβ-Ser50 (Fig. 3*F* and Table S4).

Interactions with the Q9 α2 helix involved residues from both the TCR α- and β-chains. The side-chains of the framework residue TCRα-Lys54 and Q9-Glu154 were positioned within a distance consistent with a potential salt-bridge, whereas TCRα-Arg68 was within hydrogen bonding distance of Q9-Gln166 (Fig. 3*G* and Table S4). In addition, the Q9-Lys155 side-chain was positioned within hydrogen bonding distance of the side-chain hydroxyl of TCRα-Ser31 in CDR1α and the main-chain carbonyl of TCRβ-Ser97 in CDR3β, consistent with a possible hydrogen bonding network (Fig. 3*G* and Table S4). Q9-Thr163 was similarly positioned near CDR1α, within hydrogen bonding distance of the TCRα-Asn30 side-chain and the main-chain carbonyl of TCRα-Leu29 (Fig. 3*G* and Table S4).

These interactions demonstrate that TCR C3K engages the Q9 α1 and α2 helices through a combination of germline-encoded CDR residues, CDR3β, and TCR framework residues.

### Receptor-facing sequence variation distinguishes the Q9 α2 helix from other murine MHC-I molecules

To assess the conservation of Q9 residues contacted by TCR C3K, we compared the corresponding positions across related murine MHC-I molecules. Gln72 and Glu154 were completely conserved across the MHC-I molecules examined, whereas Gln65 was conserved in all molecules except H2-D^d^, which contained Arg at this position (Table 3).

**Table 3:** Sequence conservation of Q9 residues implicated in TCR C3K recognition across murine MHC-I molecules

MHC-I	65	72	154	155	163	166
H2-Q9	Q	Q	E	K	T	Q
H2-Q7	Q	Q	E	K	T	Q
H2-Q10	Q	Q	E	**Y**	T	**E**
H2-D^b^	Q	Q	E	**H**	**E**	**E**
H2-D^d^	**R**	Q	E	**R**	**E**	**E**
H2-K^b^	Q	Q	E	**R**	**E**	**E**
H2-K^d^	Q	Q	E	**Y**	**E**	**E**
H2-K^k^	Q	Q	E	**R**	**E**	**E**
H2-L^d^	Q	Q	E	**Y**	**E**	**E**

Residues that differ from the corresponding Q9 amino acid at each position are indicated in bold.

Greater sequence variation was observed at positions 155, 163, and 166. Lys155 was restricted to H2-Q9 and H2-Q7, whereas the other MHC-I molecules contained Arg, His, or Tyr (Table 3). Of these substitutions, Arg is the most chemically conservative and may therefore be less disruptive to the C3K–Q9 interface than His or Tyr. Thr163 was conserved among H2-Q9, H2-Q7, and H2-Q10, whereas the classical MHC-I molecules contained Glu at this position (Table 3). The differences in size and charge between Thr and Glu could alter the local geometry and electrostatic environment of the TCR–pMHC interface. Similarly, Gln166 was restricted to H2-Q9 and H2-Q7, whereas the other molecules contained Glu (Table 3). Thus, several TCR C3K-contacting residues on the Q9 α2 helix differ among murine MHC-I molecules.

More broadly, comparison of the α1 and α2 helices revealed greater sequence variation within the α2 helix among the murine MHC-I molecules examined (Fig. S9). The distribution of this variation also differed according to side-chain orientation and structural function. Variable residues with receptor-accessible side-chains were prominent on the α2 helix, including Arg145, Lys155, Thr163, Gln166, Glu173, Leu174, and Lys176 (Fig. S9). In contrast, several variable residues on the α1 helix, including Glu63, Ile66, His70, Ser73, Ser77, and Thr80, had side-chains directed toward the peptide-binding groove (Fig. S9). Variable groove-facing residues were also present on the α2 helix, including Ile152, Asp156, and Ser167 (Fig. S9).

Ser167 was a distinctive feature of Q9 and Q7, whereas the other MHC-I molecules examined contained Trp at this position (Fig. S9). The smaller side-chain of Ser167 is associated with greater longitudinal opening of the Q9 peptide-binding groove. In addition, the VP2.139 main-chain at P5 was displaced toward the α2 helix relative to peptides bound to other murine MHC-I molecules (Fig. S6), adjacent to Q9-His70, which is not conserved among the murine MHC-I molecules examined (Fig. S9).

Together, these comparisons indicate that sequence variation in Q9 occurs at both receptor-accessible and groove-facing positions. Several variable receptor-accessible residues lie within the TCR C3K-binding surface, whereas variation at groove-facing positions corresponds to structural differences in the Q9 peptide-binding platform.

### Q9/VP2.139 recognition induces local conformational rearrangements in TCR C3K

We next examined whether ligand recognition was accompanied by conformational rearrangement of TCR C3K. To do this, we determined the crystal structure of unbound TCR C3K at 2.01 Å resolution (Fig. 4*A* and Table 2). Comparison of the pMHC-bound and unbound TCRs revealed little change in the overall Vα–Vβ domain arrangement, with an interdomain distance difference of only 0.4 Å (Fig. 4*A*). Thus, Q9/VP2.139 binding did not induce substantial reorientation of the TCR C3K variable domains.

Although the global TCR architecture was preserved, individual CDR loops showed greater conformational variability. The mean root mean square deviation (RMSD) across the variable domains was 0.82 ± 0.02 Å, whereas the CDR3α, CDR1β, and CDR3β loops exhibited RMSD values of 1.34 ± 0.77 Å, 0.96 ± 0.37 Å, and 0.89 ± 0.27 Å, respectively. The CDR3α and CDR3β loops also differed between the unbound TCR C3K molecules, consistent with intrinsic flexibility in these regions (Fig. 4*B*). CDR1β likewise displayed conformational variability. Although CDR1β does not directly contact Q9/VP2.139, its rearrangement may nevertheless contribute indirectly to ligand recognition by accommodating or energetically supporting local conformational changes within the TCR.

The most pronounced ligand-associated rearrangement occurred in the CDR3α loop. Upon Q9/VP2.139 engagement, residues Ala94–Leu95–Gly96 adopted a 3_10_-helical conformation (Fig. 4*B*). To determine whether this rearrangement was accompanied by changes in the ligand itself, we compared Q9/VP2.139 structures in the presence and absence of TCR C3K. This analysis revealed no substantial change in peptide presentation, suggesting that recognition is accommodated primarily by local rearrangement of the TCR rather than remodeling of the peptide–MHC surface.

These findings indicate that Q9/VP2.139 recognition preserves the overall TCR C3K variable domain architecture while being accompanied by local rearrangements of flexible CDR loops, including CDR1β, CDR3α, and CDR3β. Although the functional contribution of individual loop rearrangements cannot be established from the structural comparisons alone, the formation of a 3_10_-helical conformation in CDR3α and the accompanying local CDR variability are consistent with an induced-fit element for TCR C3K recognition.

### CDR3 loops dominate TCR C3K recognition of VP2.139

Within the C3K–Q9/VP2.139 structure, the upward-facing side-chain of P1-His was positioned adjacent to the main-chain of TCRα-Gly92 within the CDR3α loop (Fig. 5*A* and Table S5), providing local steric and van der Waals complementarity at the peptide–CDR3α interface.

**Figure 5:**
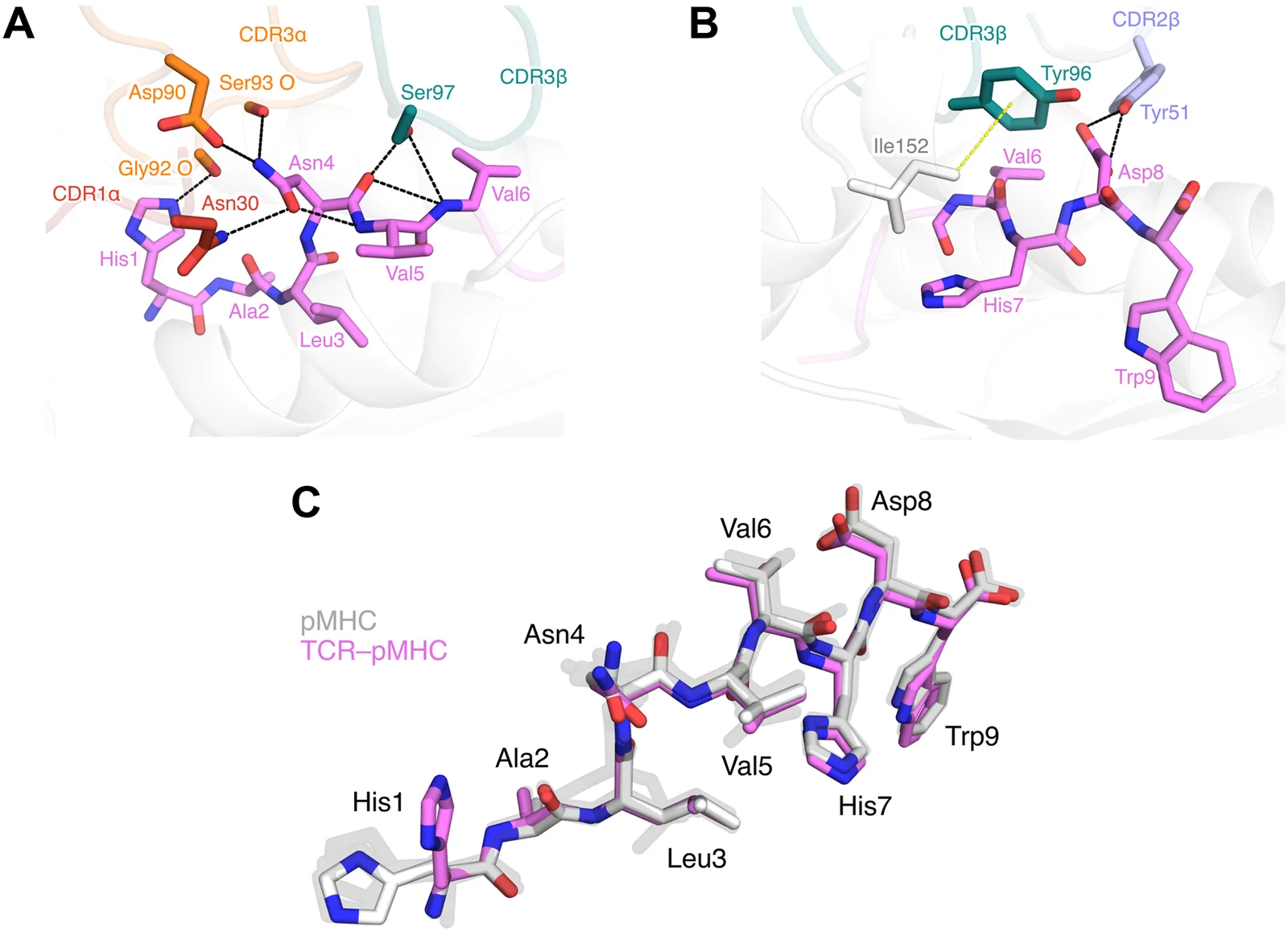
**Structural and functional mapping of VP2.139 residues contributing to TCR recognition.** Close-up views of the C3K–Q9/VP2.139 interface for peptide positions (*A*) P1–P6 and (*B*) P6–P9. Key side-chain interactions between the peptide and the TCR CDR loops are shown in *stick* representation. The CDR loops, 1α, 3α, 2β, and 3β are colored in *vermillion*, *orange*, *light blue*, and *dark teal*, respectively. Q9 and the VP2.139 peptide are colored in *light gray* and *pink*, respectively. The MHC residues defining the peptide-binding groove are shown as *sticks*, with hydrogen bonds and π-interactions indicated by *black* and *yellow dashed lines*, respectively. (*C*) Structural superposition of the VP2.139 peptide presented by Q9 bound to TCR C3K (*pink*) or unbound (*white*). Alanine mutants are shown as semitransparent *gray*. Superpositioning was performed using the MHC heavy-chain platform. CDR, complementarity-determining region; MHC, major histocompatibility complex; TCR, T-cell receptor.

P4-Asn was centrally positioned within the interface, with its side-chain lying within hydrogen bonding distance of TCRα-Asp90 and the main-chain of TCRα-Ser93 in the CDR3α loop. In addition, P4-Asn was also within contact distance of TCRα-Asn30 in CDR1α and the main-chain of TCRβ-Ser97 in the CDR3β loop (Fig. 5*A* and Table S5). Thus, P4-Asn bridges contacts involving germline-encoded and CDR3 residues at the center of the TCR–peptide interface.

In contrast, adjacent P5-Val and P6-Val residues appeared to contribute primarily through shape complementarity. Their side-chains occupied the TCR–peptide interface and were positioned within van der Waals contact distance of TCRβ-Tyr96 and TCRβ-Ser97 in the CDR3β loop (Fig. 5, *A* and *B* and Table S5). TCRβ-Ser97 may further contribute to stabilization of the interface, as its side-chain was within hydrogen bonding distance of the peptide main-chain at P5-Val and P6-Val (Fig. 5*A* and Table S5).

By comparison, residues at the peptide C-terminus appeared to make more limited direct contributions to TCR binding, although P8-Asp was positioned within hydrogen bonding distance of TCRβ-Tyr51 in the CDR2β loop (Fig. 5*B* and Table S5).

Together, these interactions suggest that TCR C3K recognition of VP2.139 is dominated by an extensive hydrogen bonding network involving the CDR3α and CDR3β loops, with additional contacts from germline-encoded CDR1α and CDR2β residues further supporting the interface.

### Alanine-scanning reveals a shared dependence on VP2.139 residues P1–P7

To determine how individual VP2.139 residues contribute to TCR recognition energetically, we performed systematic alanine-scanning across the peptide (excluding P2 and P9) and measured the binding of each Q9/VP2.139 variant to TCRs C3K and AH1 by SPR. Alanine substitution at His1, Leu3, Asn4, Val5, Val6, or His7 abolished the C3K response (Table 1). Similarly, AH1 binding was lost following alanine substitution at the same peptide positions, indicating a shared dependence on residues spanning P1–P7 of VP2.139 (Table 1).

Several of these effects were consistent with the interaction geometry observed in the C3K–Q9/VP2.139 structure. At P4, substitution of Asn with Ala removes the side-chain amide group involved in potential polar interactions with residues in and around CDR3α (Fig. 5*A*). At P5 and P6, replacement of Val with the smaller Ala side-chain reduces the side-chain surface available for packing against TCRβ-Tyr96 and TCRβ-Ser97 and would therefore reduce local shape complementarity (Fig. 5, *A* and *B*). Similarly, P1A removes the larger His side-chain positioned adjacent to CDR3α-Gly92, reducing the available surface for packing at the peptide–CDR3α interface (Fig. 5*A*).

In contrast, alanine substitution at Asp8 had minimal impact on the measured binding affinity of either TCR (Table 1). Thus, although P8-Asp is positioned adjacent to TCRβ-Tyr51 in a geometry compatible with a polar interaction, removal of the Asp side-chain produced little measurable energetic penalty (Fig. 5*B*). This result illustrates that a structurally apparent contact does not necessarily represent a major energetic determinant of binding.

To assess whether loss of TCR binding reflected altered peptide presentation rather than loss of direct energetic contacts, we determined crystal structures of Q9/VP2.139 variants P1A (AALNVVHDW), P4A (HALAVVHDW), P5A (HALNAVHDW), and P8A (HALNVVHAW) (Figs. 5*C*, S5, and S10). Superposition of these structures with WT Q9/VP2.139 revealed low peptide RMSDs of 0.437 Å for P1A, 0.105 Å for P4A, 0.148 Å for P5A, and 0.249 Å for P8A. For comparison, VP2.139 in the C3K–Q9/VP2.139 complex exhibited an RMSD of 0.193 Å relative to the unliganded Q9/VP2.139 structure. All peptide RMSDs were therefore <0.5 Å, demonstrating that these substitutions did not substantially alter the overall conformation of the peptide within the Q9 groove. These analyses demonstrated that differences in TCR affinity for variants P1A, P4A, P5A, and P8A could not be attributed to gross perturbations in peptide presentation by Q9.

Collectively, the alanine-scanning and structural analyses identify VP2.139 residues P1–P7 as the principal energetic determinants of TCRs C3K and AH1 binding, providing further support for a CDR3-centered mode of Q9-restricted TCR recognition.

### Structural features of Q9 predict impaired CD8 engagement

To identify structural features that may underlie reduced CD8 coreceptor engagement by Q9, we compared the Q9 α3-domain with that of the classical murine MHC-Ia molecule H2-K^b^ in complex with CD8αα (Protein Data Bank [PDB]: 1BQH (18)). Superposition of the MHC α3-domains showed that Q9 and H2-K^b^ retain a conserved immunoglobulin-like fold, but revealed pronounced differences at the CD8-binding surface (Fig 6, *A–C*).

**Figure 6:**
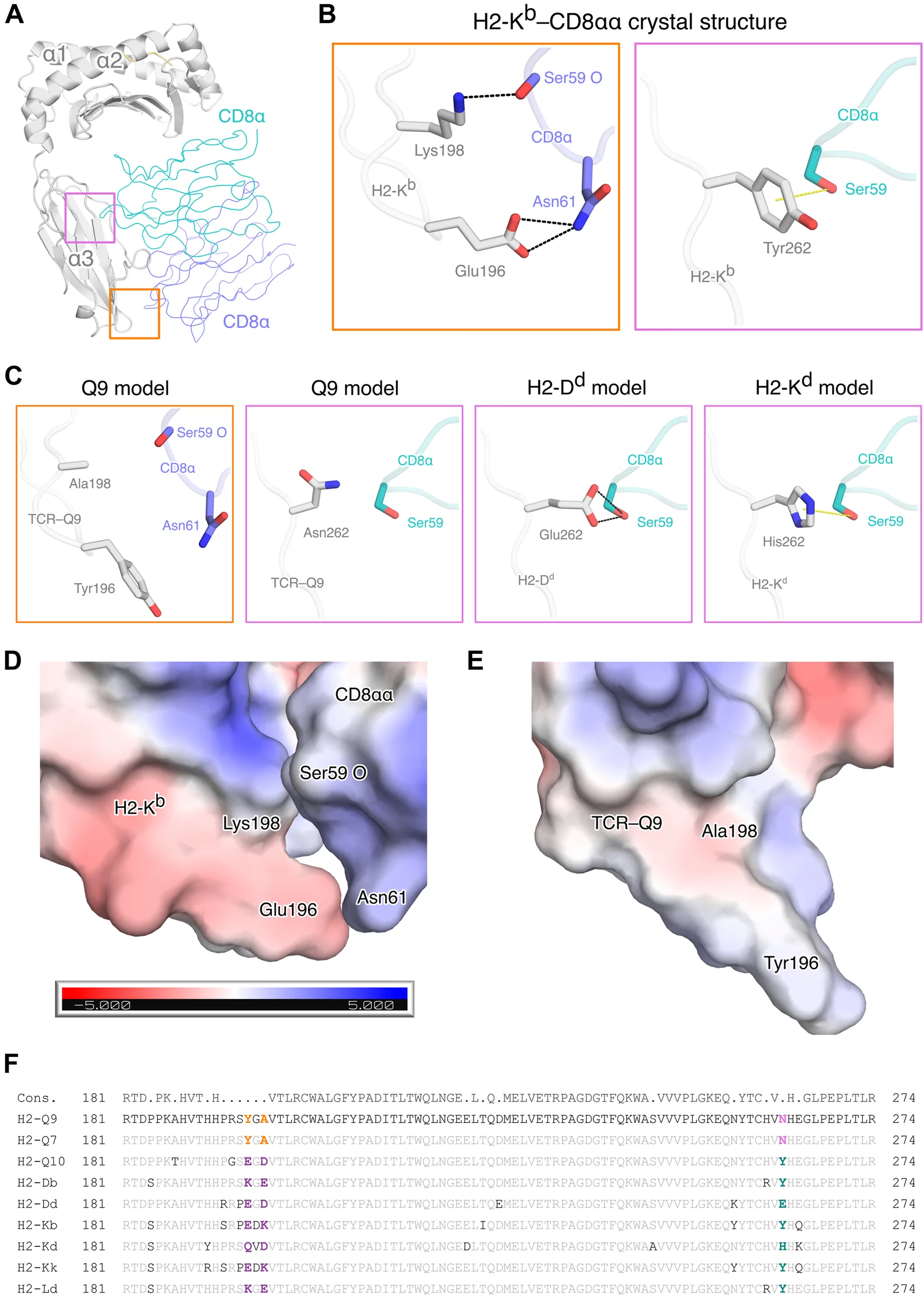
**CD8αα-binding features of Q9 compared with murine MHC-Ia molecules.** (*A*) Cartoon representation of H2-K^b^ (*light gray*) in complex with CD8αα (*blue* and *teal*) shown as *ribbon* diagrams, derived from PDB: 1BQH. The MHC heavy-chain α1-, α2-, and α3-domains are indicated. *Orange* and *pink* boxes denote the corresponding regions of the MHC α3-domain examined throughout the figure. (*B*) Close-up views of the H2-K^b^–CD8αα interface surrounding MHC heavy-chain residues 196 and 198 (*left*) and residue 262 (*right*). Key MHC–CD8αα interactions are shown as *sticks*. Hydrogen bonds and π-interactions are indicated by *black* and *yellowdashed lines*, respectively. (*C*) Structural models generated by superposition of the MHC α3-domains. H2-D^d^ and H2-K^d^ structures were derived from PDB: 1BII and 5GSR, respectively. Adaptive Poisson-Boltzmann Solver (APBS) electrostatic surface potential of (*D*) H2-K^b^ (*left*) and CD8αα (*right*), and (*E*) Q9, illustrating differences in charge distribution at the binding interface. Electrostatic potentials are displayed using a color scale ranging from −5 to +5 kT/e, with negative, neutral, and positive electrostatic potentials represented by *red*, *white*, and *blue*, respectively. (*F*) Sequence alignment of the α3-domain of the known surface expressed Qa-2 family (H2-Q7 and H2-Q9), Qa-1^b^ (H2-Q10), and classical murine MHC-Ia molecules. The consensus sequence is shown above the alignment. Nonpolar substitutions are highlighted in *orange*, charged or polar substitutions in *purple*, and position 262 residues predicted from modeling to contact CD8αα are highlighted in *teal*, whereas noncontacting residues are highlighted in *pink*. MHC, major histocompatibility complex; PDB, Protein Data Bank.

In the H2-K^b^–CD8αα complex, CD8αα binding is supported by a charged interface. Specifically, the main-chain carbonyl of CD8αα-Ser59 and the side-chain of CD8αα-Asn61 form hydrogen bonds with H2-K^b^-Glu196 and H2-K^b^-Lys198, respectively, contributing to electrostatic complementarity between H2-K^b^ and the coreceptor (Fig. 6*B*). In Q9, the corresponding positions are occupied by Q9-Tyr196 and Q9-Ala198 (Fig. 6*C*). These substitutions replace charged residues with polar or nonpolar residues, thereby reducing the charged character of the equivalent α3-domain surface relative to H2-K^b^.

Consistent with this interpretation, electrostatic surface analysis showed that the H2-K^b^ α3-domain presents a charged surface compatible with CD8αα engagement (Fig. 6*D*), whereas the corresponding region of Q9 is substantially neutralized (Fig. 6*E*). In Q9, this loss of electrostatic complementarity is accompanied by altered local topology and exposure of a more hydrophobic surface within the predicted CD8-binding region. Together, these changes would be expected to disfavor interaction with the predominantly charged CD8αα interface.

Additional differences were observed at Q9 position 262, which lies at the CD8-binding interface. In the H2-K^b^–CD8αα complex, H2-K^b^-Tyr262 forms a π-interaction involving the side-chain hydroxyl of CD8αα-Ser59 (Fig. 6*B*). To compare the alternative residues found at this position in classical MHC-Ia molecules, the experimentally determined α3-domain structures of H2-D^d^ (PDB: 1BII (19)) and H2-K^d^ (PDB: 5GSR (20)) were superimposed onto the H2-K^b^ α3-domain in the H2-K^b^–CD8αα complex. In the H2-D^d^ model, Glu262 could preserve contact with the CD8αα-Ser59 side-chain hydroxyl through hydrogen bonding (Fig. 6*C*). Similarly, His262 in the H2-K^d^ model retained the potential for a π-interaction with CD8αα-Ser59 (Fig. 6*C*). In contrast, Q9-Asn262 did not support an equivalent interaction, likely because of its shorter side-chain (Fig. 6*C*).

Next, we compared the sequence alignment of the murine MHC-I α3-domains, which further supported the structural comparison. Residues at positions 196 and 198 are conserved as charged residues among classical murine MHC-Ia molecules that bind CD8, whereas Q9 and related Qa-2 family members encode neutral or nonpolar residues at these sites (Fig. 6*F*). Overall, these findings suggest that Qa-2 family molecules lack several structural features that support CD8 engagement by classical MHC-Ia molecules, including charged residues at positions 196 and 198, and a residue at position 262 capable of stabilizing interactions with CD8.

## Discussion

Nonclassical MHC-Ib molecules broaden antigen presentation beyond the highly polymorphic classical MHC-Ia system, yet the molecular rules governing their recognition by conventional αβ TCRs remain less well defined (1). Here, we provide a structural framework for understanding how the TCR C3K recognizes the polyomavirus-derived VP2.139 peptide presented by the nonclassical MHC-Ib Q9.

By combining crystallographic, biophysical, and mutational analyses, we show that Q9/VP2.139 is recognized through a highly peptide-centric and cooperative interface. Structural comparison of the Q9 α3-domain further suggests features that may contribute to reduced CD8 binding.

One finding of this study is the strong energetic dominance of the peptide in Q9-restricted TCR recognition. Namely, alanine substitution across positions P1–P7 abolished binding by both TCRs C3K and AH1. This contrasts with interfaces in which a smaller number of peptide residues dominate binding energetics (21, 22, 23), and instead suggests that multiple peptide contacts act in parallel to stabilize the TCR–pMHC complex. The preservation of peptide presentation in structures of Q9-bound alanine-substituted VP2.139 peptides further indicates that loss of binding is attributable primarily to disruption of TCR-recognition determinants rather than gross remodeling of the Q9-bound peptide.

The distinct genetic profiles of TCRs C3K and AH1 further suggest that Q9/VP2.139 imposes a restricted energetic solution on different TCRs. Although these TCRs use different Vα and Vβ gene segments, both depend on an overlapping energetic core within VP2.139. A comparable convergence on conserved energetic features has been observed among structurally distinct HLA-E-restricted TCRs, indicating that nonclassical pMHC surfaces can direct different receptors toward shared recognition solutions (24). In the case of Q9, the previously reported Vβ bias among Q9-restricted T-cells may therefore reflect selection for receptors capable of accommodating the composite Q9/peptide surface rather than an obligatory germline-encoded interaction with Q9. Consistent with this interpretation, the TCR C3K interface is dominated by somatically generated CDR3 residues rather than extensive contacts mediated by a particular germline-encoded Vβ loop.

Despite this peptide-focused recognition, TCR C3K engages Q9/VP2.139 with the diagonal polarity characteristic of classical MHC-Ia-restricted TCRs (15, 25). The nonclassical function of Q9 therefore does not require a fundamentally unconventional docking geometry. Instead, TCR C3K is shifted toward the N-terminal half of VP2.139, positioning the receptor over the most solvent-exposed region of the peptide and concentrating its contacts on the peptide surface. Similar canonical docking has been observed for an antiviral HLA-E-restricted TCR, further indicating that presentation by an MHC-Ib molecule does not necessarily impose a distinct TCR-binding orientation (26). Thus, Q9 appears to preserve the overall topology of conventional TCR–pMHC recognition while altering the relative contributions of peptide, MHC, and coreceptor engagement.

Our data also provide a structural framework for understanding the reduced dependence of Q9-restricted T-cells on CD8 coreceptor engagement. Residues within the Q9 α3-domain that are typically conserved in classical MHC-Ia molecules and contribute to CD8 binding are absent, resulting in reduced charge and altered surface topology (18). These features are consistent with diminished electrostatic complementarity and may impair productive CD8 interactions, although this has not been directly measured. It is therefore likely that Q9 supports reduced, rather than absent, coreceptor engagement. In this context, the relatively large interface area and extensive peptide contacts observed for TCR C3K may partially compensate by increasing intrinsic TCR–pMHC binding stability.

The relevance of TCR C3K as a model receptor is further supported by prior *in vivo* retrogenic studies demonstrating specific recognition of the Q9/VP2.139 epitope during MuPyV infection (17). Thus, the structural and mutational findings reported here can be interpreted in the context of an authentic antiviral response.

The biological implications of Q9-mediated antigen presentation extend to tumor immunity. Qa-2 expression has been associated with increased susceptibility of murine tumors to immune-mediated clearance, whereas loss of Q8/Q9 expression is frequently observed in tumor cell lines and appears to occur early during tumor progression (27, 28). This contrasts with human HLA-G, a MHC-Ib molecule often upregulated in tumors and associated with immune evasion (28, 29, 30, 31). These observations suggest that, despite some structural and evolutionary parallels, Qa-2 molecules and HLA-G may play fundamentally different roles in immune regulation. In this context, the ability of Q9 to support peptide-centric TCR recognition may contribute to effective immunosurveillance, and loss of Q9 expression may represent a mechanism of immune escape.

In summary, Q9 presents a conventionally processed viral peptide antigen but is recognized through a highly cooperative and peptide-driven TCR interface assembled on a MHC-Ib scaffold. Altered α3-domain features may reduce the contribution of CD8 coreceptor engagement. Together, these findings highlight the flexibility of the TCR–pMHC recognition system and suggest that MHC-Ib molecules can support effective CD8^+^ T-cell responses through mechanisms that diverge quantitatively, rather than qualitatively, from classical paradigms.

## Experimental procedures

### Generation of Q9/VP2.139-restricted TCRs

TCR sequences were obtained from Q9/VP2.139-specific CD8^+^ T-cell hybridomas provided by Dr Aron E. Lukacher. The hybridomas were generated by fusion of a MuPyV-specific K^b−/−^D^b−/−^ CD8^+^ T-cell clone with a CD8-transfected BW5147 fusion partner, as previously described (17). TCR gene usage and CDR3 sequences are provided in the Table S1.

### Protein expression and purification

The extracellular-domains of Q9, murine β-2-microglobulin (β_2_M), and the α-chain and β-chain of TCRs C3K and AH1 were cloned into the pET-30a(+) vector and expressed as inclusion bodies within *Escherichia coli* BL21(DE3) cells. Inclusion bodies were harvested and solubilized in 20 mM Tris–HCl pH 8.0, 6 M guanidine hydrochloride, 1 mM EDTA, 1 mM DTT, and 0.2 mM PMSF. Q9 heavy-chain was refolded in the presence of murine β_2_M (90 mg and 20 mg, respectively) and 5 mg of the MuPyV VP2.139 peptide (amino acid sequence: HALNVVHDW). C3K and AH1 were refolded separately at a 1:1 ratio of TCR α- and β-chains.

Refolds were carried out in 0.5 L refold buffer (0.1 M Tris–HCl pH 8.0, 2 mM EDTA, 0.4 M L-Arginine HCl, 0.5 mM oxidized glutathione, 5 mM reduced glutathione, and 0.2 mM PMSF) for 2 days. The refold solution was then dialyzed into 10 mM Tris–HCl pH 8.0. Refolded protein was initially purified by diethylaminoethyl (DEAE)-cellulose anion exchange followed by size-exclusion chromatography in 10 mM Tris pH 8.0 and 150 mM NaCl. Refolded TCRs were subjected to an additional purification step involving hydrophobic interaction chromatography with a HiTrap Phenyl HP in 0.8 M (NH_4_)_2_SO_4_ for removal of β-chain homodimer. The final purification step was anion exchange chromatography with a HiTrap Q column with a linear gradient of 10 mM Tris pH 8.0, 0 to 1 M NaCl. Samples were buffer exchanged to 10 mM Tris pH 8.0, 150 mM NaCl for storage prior to use.

TCR C3K was co-complexed with equal stoichiometric amounts of Q9/VP2.139 and incubated for 30 min at room temperature prior to purification by size-exclusion chromatography.

### SPR affinity measurements

A Biacore T200 was used for SPR affinity measurement. Two independent experiments using different batches of protein were performed at 298 K. The buffer used contained 20 mM Hepes pH 7.5, 300 mM NaCl, and 0.005% surfactant P20 (HBS-300-P20). The Q9/VP2.139 or alanine mutant was biotinylated and immobilized on a streptavidin-coated sensor chip (Biacore) to achieve appropriate surface densities. TCRs C3K and AH1 at 200 μM were serially diluted in HBS-300-P20 and passed simultaneously over the test (Q9/VP2.139) and empty flow cell surfaces at a flow rate of 5 μl/min. H2-K^b^ in complex with the pBM1 peptide (amino acid sequence: INFDFNTI) was used as a negative control. No separate positive control was included, as binding to WT Q9/VP2.139 served as the reference interaction for comparison with the alanine-substituted peptide variants. Replicates 1 and 2 represent two independent SPR experiments (n = 2) performed using separate protein preparations. Within each independent experiment, each TCR sample was analyzed in duplicate. All obtained data were analyzed using steady-state equilibrium analysis with GraphPad Prism version 11.0.0 (GraphPad Software).

### Crystallization and X-ray data collection

Crystals were obtained by hanging drop experiments with C3K–Q9/VP2.139 complex (10 mg/ml) incubated over a reservoir at 293.15 K containing 0.2 M ammonium sulfate, 0.1 M Na3 Cit pH 5.6, 16% (w/v) PEG 3350. Q9/VP2.139 crystals (6 mg/ml) were grown at 293.15 K over a reservoir containing 0.2 M ammonium acetate, 0.1 M Hepes pH 7.0, 20% (w/v) PEG 3350. Prior to flash freezing in liquid nitrogen crystals were cryoprotected in reservoir solution supplemented with 20% (v/v) glycerol. All X-ray diffraction data were obtained at the Australian synchrotron MX2 and MX3 beamlines (32).

### Structure determination and refinement

Diffraction data were processed with X-ray Detector Software (XDS) (33) and then scaled and merged with AIMLESS (34). Phases were obtained by molecular replacement using PHASER (35) with Q9/L19 used as the initial search model (PDB: 1K8D (11)) followed by rounds of restrained refinement in PHENIX (36) and model building with COOT (37). A single round of simulated annealing was performed to reduce model bias, and Translation–Libration–Screw (TLS) refinement was applied during the final stages of refinement. Data collection and refinement statistics for C3K–Q9/VP2.139 complex and binary structures are summarized in Table 2. Data collection and refinement statistics for VP2.139 alanine variants bound to Q9 are summarized in Table 2.

### Docking and incidence angle determination

To quantify the orientation of the TCR relative to the pMHC complex, TCR crossing and incidence angles were calculated using TCR3d (38), according to the geometric definitions implemented by the program for TCR–pMHC complexes. Angles were determined for the C3K–Q9/VP2.139 complex characterized in this study and for a reference set of murine TCR–pMHC-I complexes. The following PDB entries were included in the comparative analysis: 1FO0 (39), 1G6R (40), 1KJ2 (41), 1MWA (42), 1NAM (43), 2OI9 (44), 2OL3 (45), 3PQY (46), 3TF7 (15), 3TFK (15), 3TPU (15), 4MVB (47), 4MXQ (47), 4N0C (47), 4N5E (47), 5IVX (48), 5M02 (49), 5WLG (50), 6G9Q (49), 6L9L (51), and 7JWJ (25). The human-derived SB27–HLA-B∗35:08/LPEP TCR–pMHC-I complex (PDB: 2AK4) was included as an additional comparator (52).

### Calculation of the buried surface area

The BSA was determined by subtracting the solvent accessible surface area (SASA) of the TCR–pMHC complex from the sum of the solvent accessible surface areas of the TCR and the pMHC (BSA = SASA_TCR_ + SASA_pMHC_–SASA_TCR–pMHC_). Calculation of the SASA was done with the program “FreeSASA” and a 1.4 Å probe radius (53).

### Calculation of peptide-binding groove volumes

The volumes of the peptide-binding grooves of murine MHC-I molecules were calculated with the program POVME 3.0 (54) using the detect command. The following configurations were used: clashing cutoff = 3.0, n neighbors = 3, n spheres = 4, pocket detection resolution = 4.0, pocket measuring resolution = 1.0, sphere padding = 1.0. Binding volumes given by the program that were inadequate were manually adjusted accordingly.

### Calculation of the electrostatic surfaces

Electrostatic potentials of the MHC-I α3-domains and CD8αα were calculated using the APBS Electrostatics plugin (55) in PyMOL (56) and mapped onto the corresponding molecular surfaces.

## Data availability

The atomic coordinates and structure factors have been deposited in the Protein Data Bank under the following accession codes: C3K–Q9/VP2.139 ternary complex, 43RH; unliganded TCR C3K, 43SB; Q9/VP2.139 binary complex, 43RZ; P1A variant, 43SK; P4A variant, 43SR; P5A variant, 43SY; P8A variant, 43TE. All other data are contained within the article and its supporting information.

## Supporting information

This article contains supporting information.

## Conflict of interest

The authors declare that they have no conflicts of interest with the contents of this article.
