# Supplementary material for: A murine MHC-Ib molecule, H2-Q9, drives peptide-centric T-cell receptor recognition of a viral antigen
Source: J Biol Chem. 2026 Sep 17;302(11):113574. doi: 10.1016/j.jbc.2026.113574 (PMC13696813; doi:10.1016/j.jbc.2026.113574)
Supplement: Supplementary file 1 [file mmc1.pdf]

Supplementary Information

A murine MHC-Ib molecule, H2-Q9, drives peptide-centric T cell receptor recognition of a viral antigen

Luke Tennant[1], Chacko Jobichen[1$], Mai T. Tran[1], Carine Farenc[1], Stephanie Gras[1%], Aron E. Lukacher[2], Lucy C. Sullivan[3], Andrew G. Brooks[3] & Jamie Rossjohn[1#]

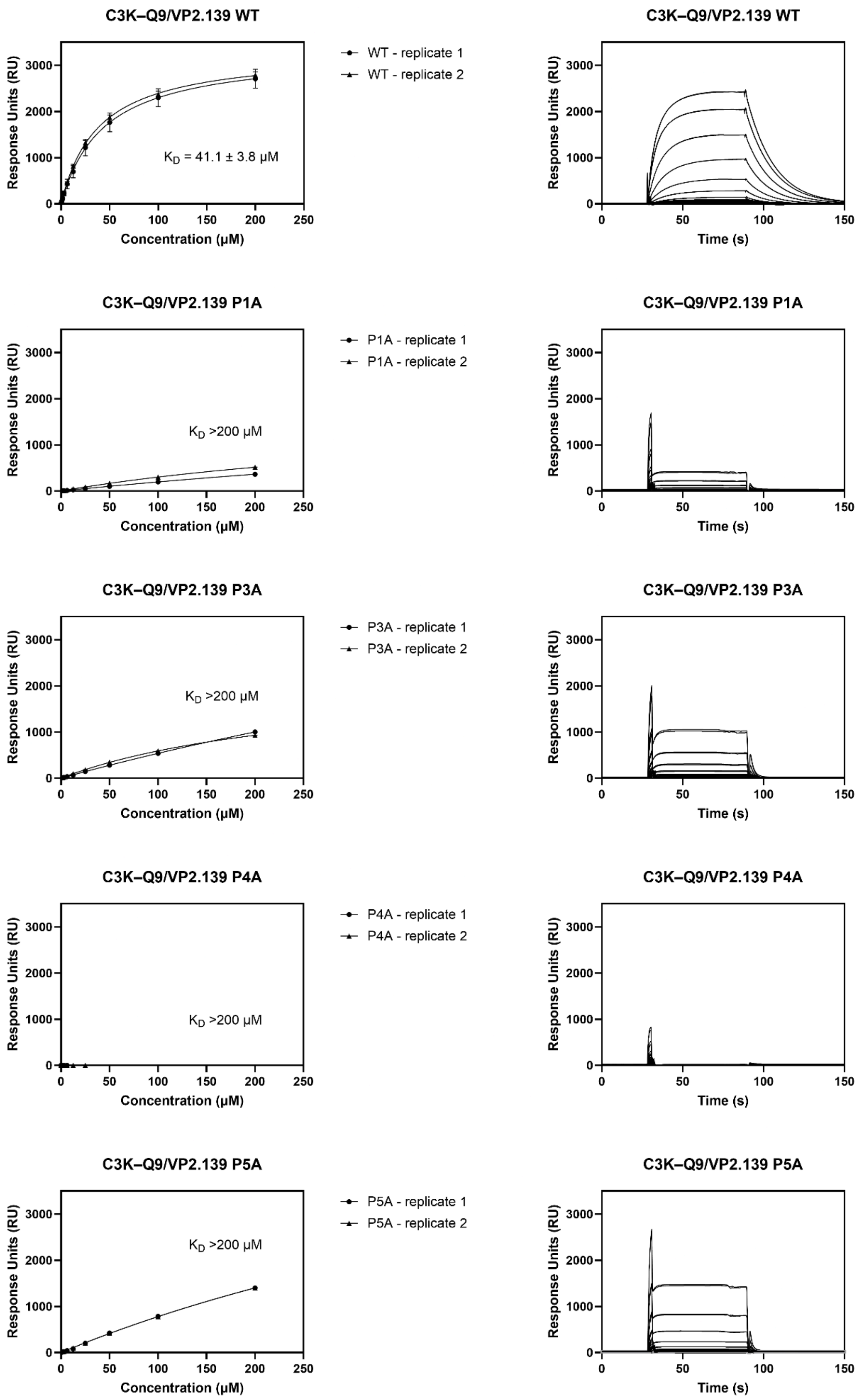

Supplementary Figure 1. Alanine scanning mutagenesis of Q9/VP2.139 at P1–P5 for TCR C3K.

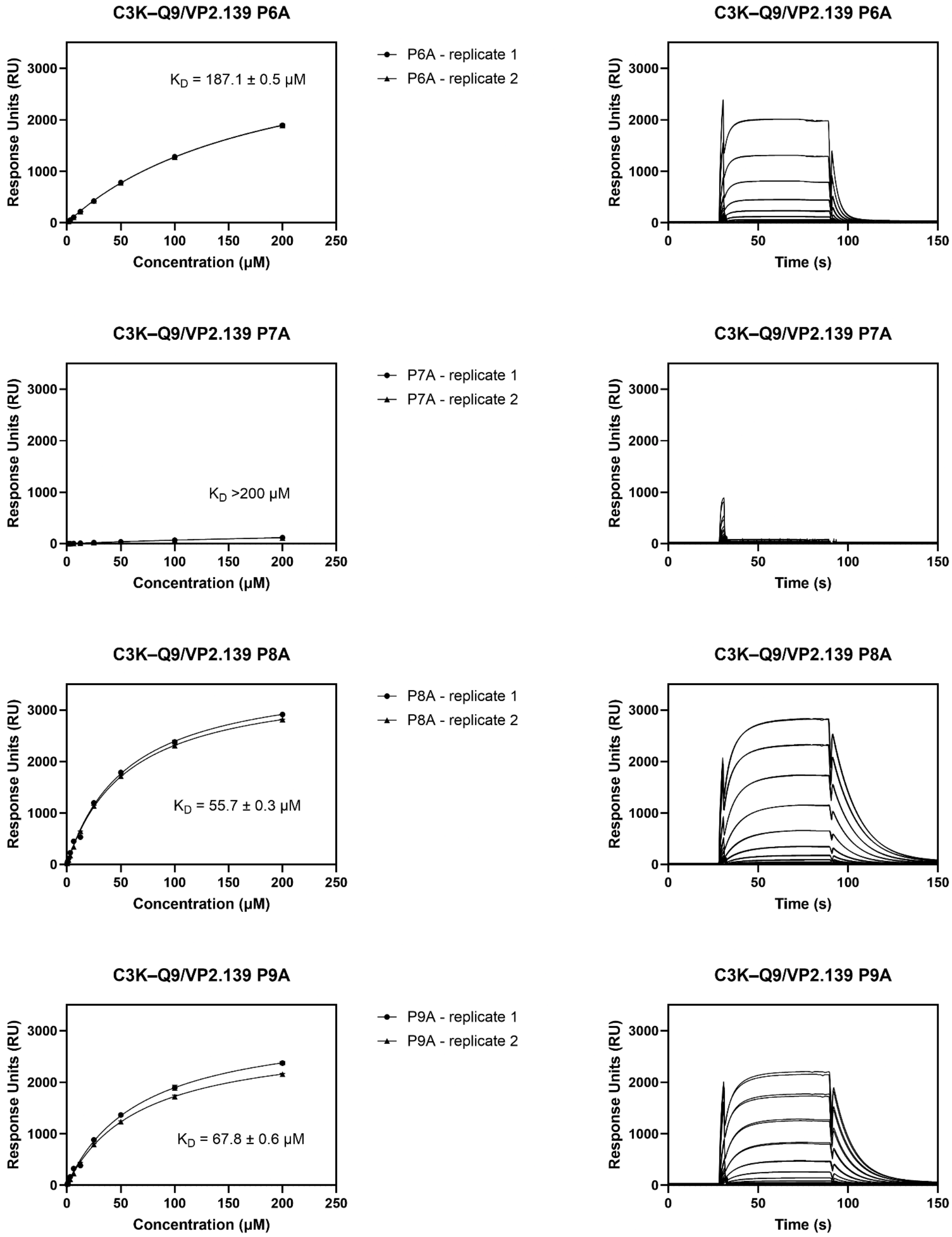

Supplementary Figure 2. Alanine scanning mutagenesis of Q9/VP2.139 at P6–P9 for TCR C3K.

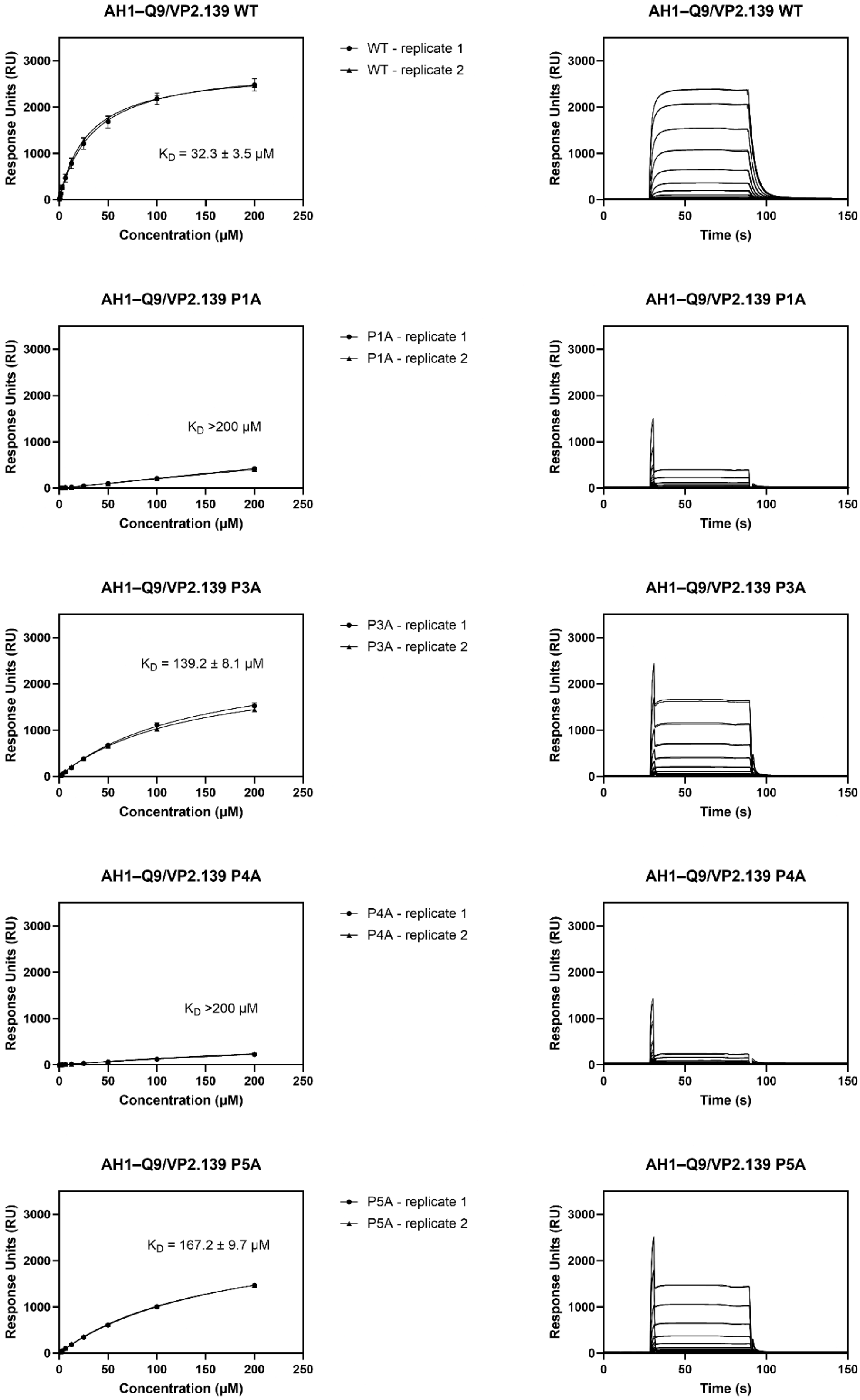

Supplementary Figure 3. Alanine scanning mutagenesis of Q9/VP2.139 at P6–P9 for TCR AH1.

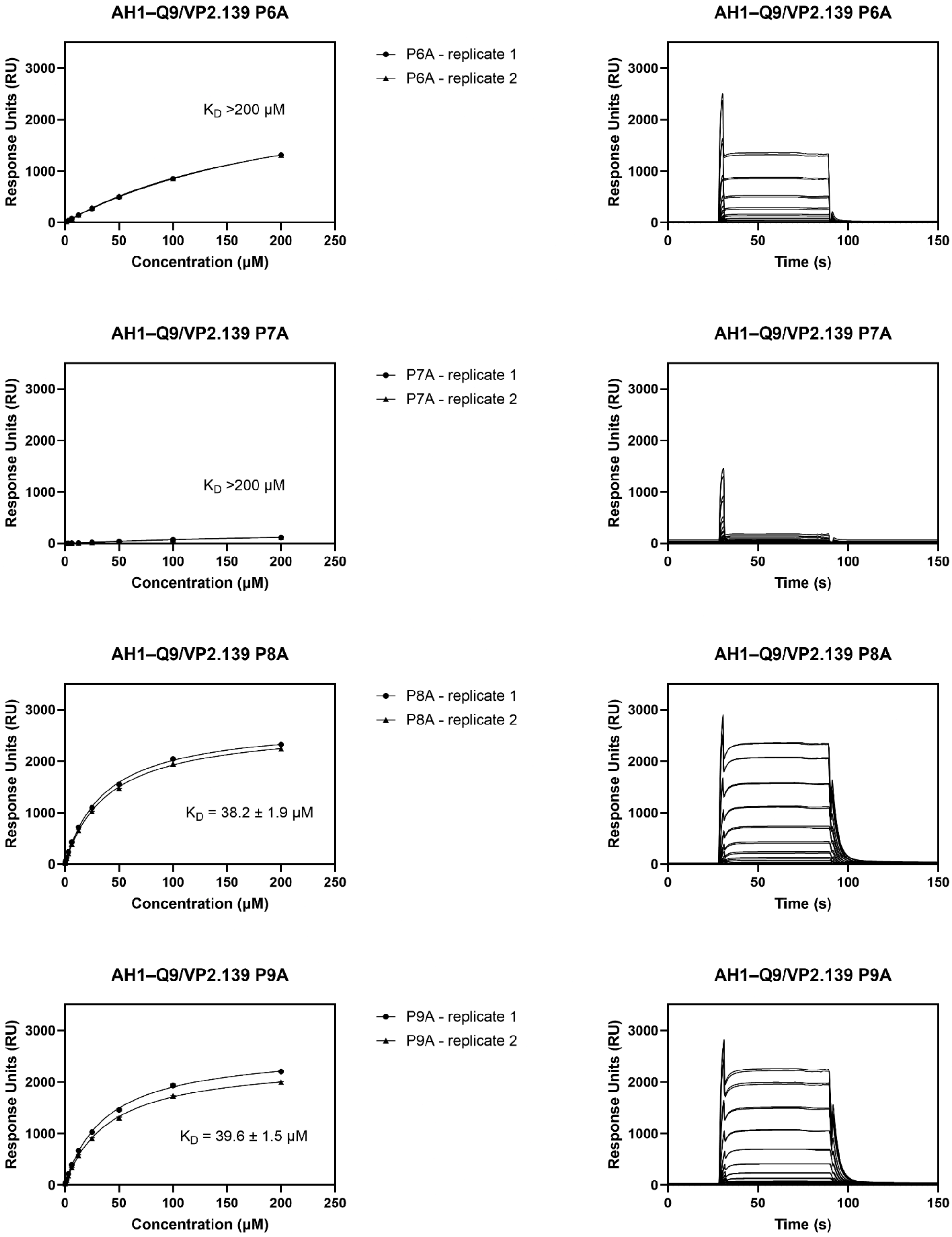

Supplementary Figure 4. Alanine scanning mutagenesis of Q9/VP2.139 at P6–P9 for TCR AH1.

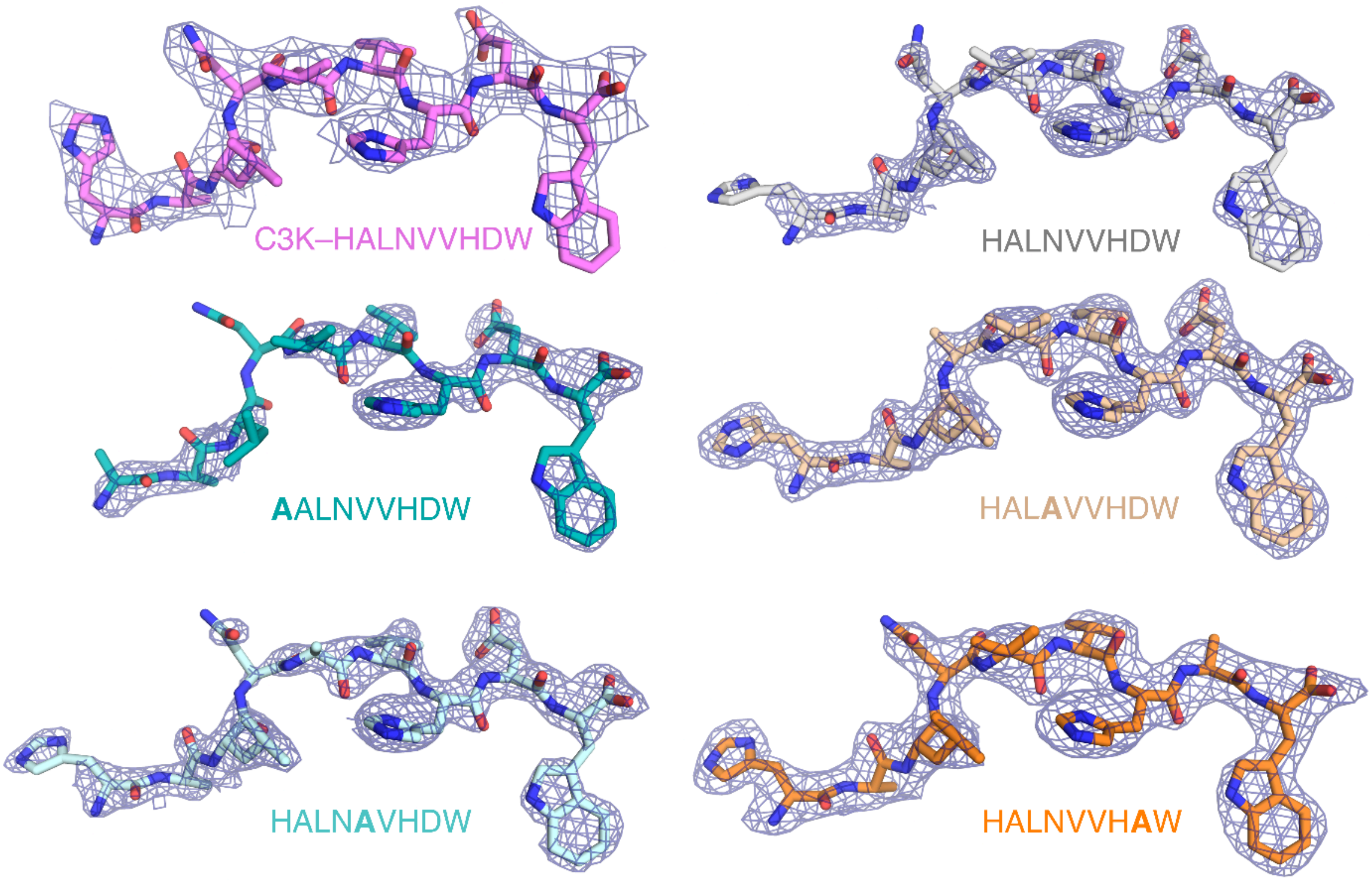

Supplementary Figure 5. Simulated-annealing OMIT electron-density maps for the TCR C3K-bound, wild-type, and alanine-substituted VP2.139 peptides. Simulated-annealing OMIT 2mFo–DFc electron-density maps are contoured at 1.0σ. Peptides are shown as sticks, and the corresponding 2mFo–DFc electron density is shown as a *blue* mesh. The C3K-bound VP2.139 peptide is coloured *pink*, the wild-type VP2.139 peptide *light grey*, P1A *teal*, P4A *wheat*, P5A *light cyan*, and P8A *orange*.

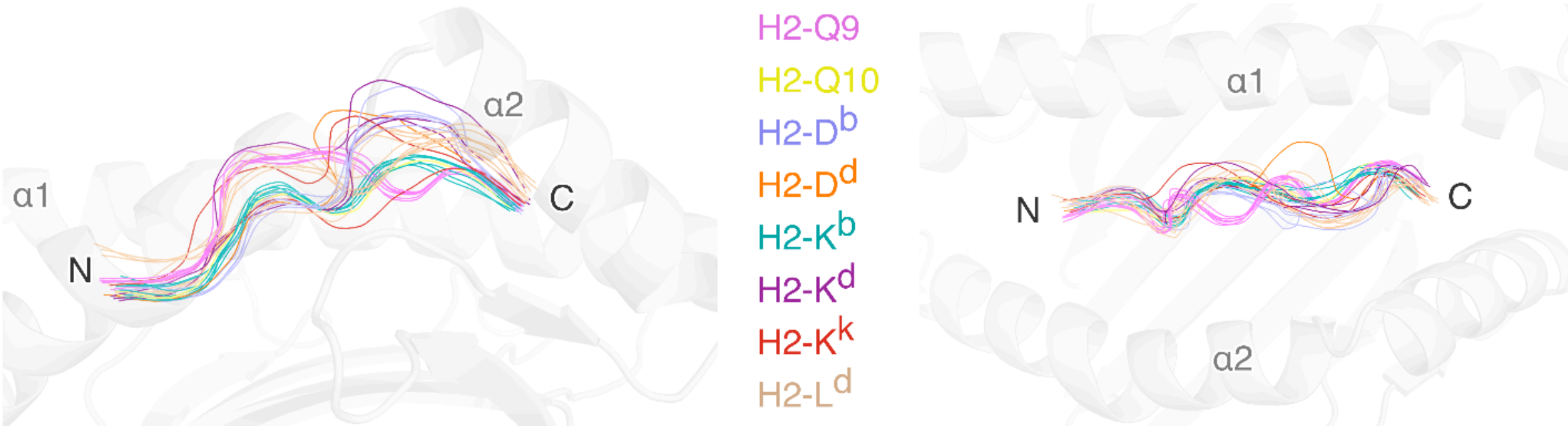

Supplementary Figure 6. Peptide conformations of murine MHC-I molecules. Superposition of the peptide-binding grooves of murine MHC-I molecules, highlighting differences in the conformations of their bound peptides. The peptide-binding grooves are shown in side view (left) and top-down view (right), with the MHC α1 and α2 helices indicated. For clarity, only the H2-Q9 MHC molecule is shown as a *light grey* cartoon. Presented peptides are shown as ribbon diagrams and coloured according to the corresponding MHC-I molecule: H2-Q9, *pink*; H2-Q10 (Qa-1^{b}), *yellow*; H2-D^{b}, *light blue*; H2-D^{d}, *orange*; H2-K^{b}, *teal*; H2-K^{d}, *purple*; H2-K^{k}, *vermillion*; and H2-L^{d}, *wheat*. PDBs used are identical to those listed for Supplementary Table 3.

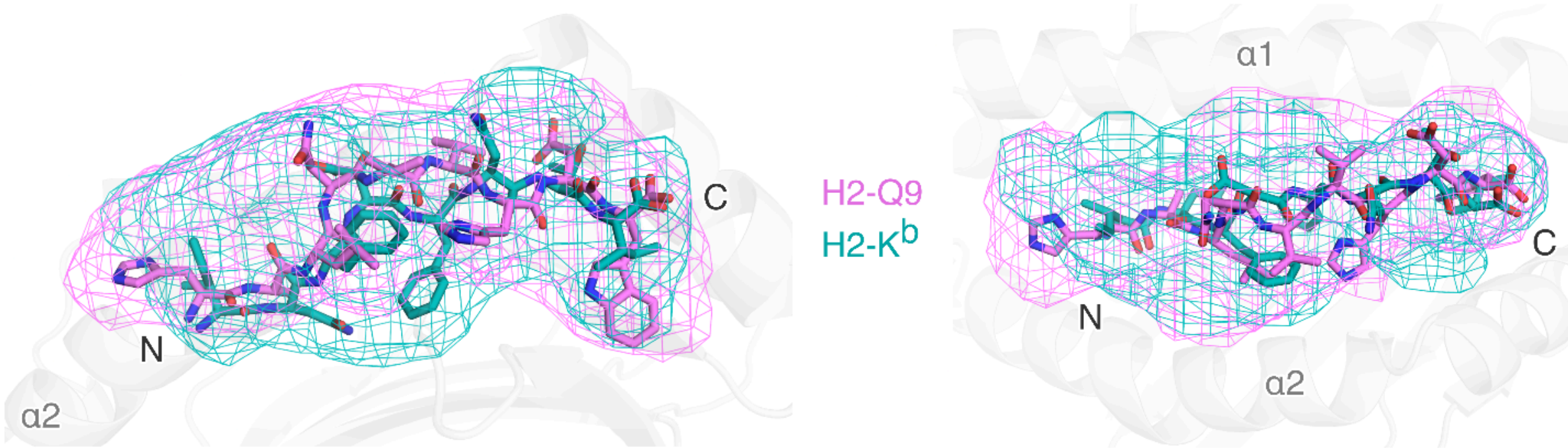

Supplementary Figure 7. Peptide-binding groove volumes of H2-Q9 and H2-K^b. Superposition of the peptide-binding grooves of H2-Q9 and H2-K^b, highlighting differences in peptide accommodation within the binding cleft. The peptide-binding grooves are shown in side view (left) and top-down view (right), with the MHC α1 and α2 helices indicated. For clarity, only the H2-Q9 MHC molecule is shown as a *light grey* cartoon. Presented peptides are shown as sticks and coloured according to the corresponding MHC-I molecule: H2-Q9, *pink*; and H2-K^b, *teal*. Peptide-binding groove volumes are shown in the corresponding colours.

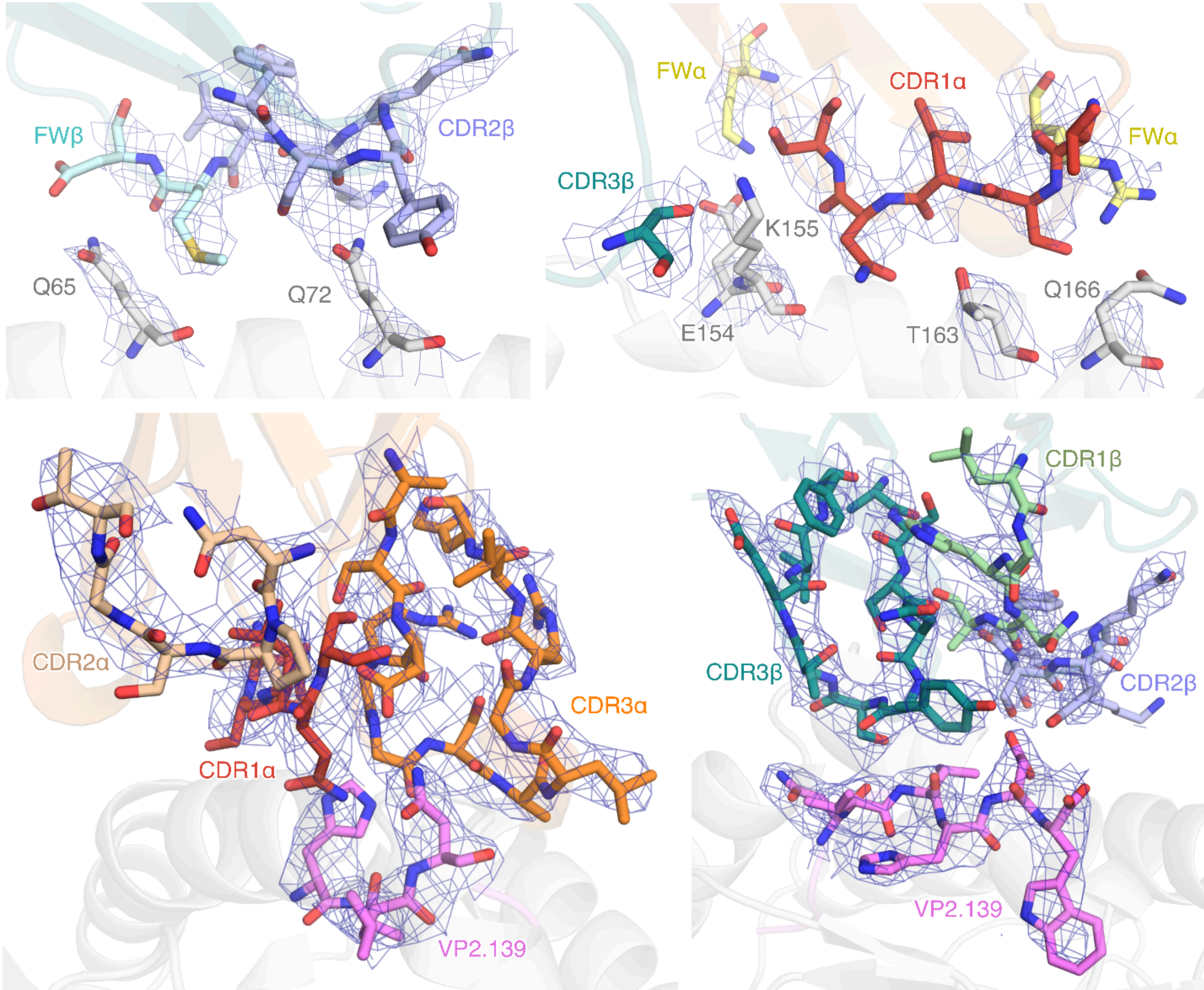

Supplementary Figure 8. Simulated-annealing OMIT electron-density maps for the C3K–Q9/VP2.139 complex. Simulated-annealing OMIT 2mFo–DFc electron-density maps, contoured at 1.0σ, are shown for key residues at the C3K–Q9/VP2.139 interface. Close-up views show interactions involving the Q9 α1 helix (top left), Q2 α2 helix (top right), TCR α-chain CDR loops (bottom left), and TCR β-chain CDR loops (bottom right). Key residues are shown as sticks, with the corresponding 2mFo–DFc electron density shown as a *blue* mesh. CDR1α, CDR2α, CDR3α, CDR1β, CDR2β, and CDR3β are colored *vermillion*, *wheat*, *orange*, *green*, *light blue*, and *dark teal*, respectively. The α- and β-chain framework (FW) regions are colored *light yellow* and *light cyan*, respectively. Q9 and the VP2.139 peptide are colored *light grey* and *pink*, respectively.

α1 helix

```
Cons.     57   PEYWE..T..AK..EQ.F...LR....YYN   86
H2-Q9     57   PEYWERETQIAKGHEQSFRGSLRTAQSYYN   86
H2-Q7     57   PEYWERETQIAKGHEQSFRGSLRTAQSYYN   86
H2-Q10    57   PEYWERETQRAKGNEQSFHVSLRTLLGYYN   86
H2-Db     57   PEYWERETQKAKGQEQWFRVSLRNLLGYYN   86
H2-Dd     57   PEYWERETRRAKGNEQSFRVDLRTALRYYN   86
H2-Kb     57   PEYWERETQKAKGNEQSFRVDLRTLLGYYN   86
H2-Kd     57   PEYWEEQTQRAKSDEQWFRVSLRTAQRYYN   86
H2-Kk     57   PEYWERNTQIAKGNEQIFRVNLRTALRYYN   86
H2-Ld     57   PEYWERITQIAKGQEQWFRVNLRTLLGYYN   86
```

α2 helix

```
Cons.    138   .AA.IT..KWEQ.G.AE...AYLE..C...LRRYL..G.   176
H2-Q9    138   MAAQITRRKWEQAGIAEKDQAYLEGTCMQSLRRYLELGK   176
H2-Q7    138   MAAQITRRKWEQAGIAEKDQAYLEGTCMQSLRRYLQLGK   176
H2-Q10   138   VAAIITRRKWEQAGAAEYYRAYLEAECVEWLLRYLELGK   176
H2-Db    138   MAAQITRRKWEQSGAAEHYKAYLEGECVEWLHRYLKNGN   176
H2-Dd    138   MAAQITRRKWEQAGAAERDRAYLEGECVEWLRRYLKNGN   176
H2-Kb    138   MAALITKHKWEQAGEAERLRAYLEGTCVEWLRRYLKNGN   176
H2-Kd    138   TAALITRRKWEQAGDAEYYRAYLEGECVEWLRRYLELGN   176
H2-Kk    138   MAALITKHKWEQAGDAERDRAYLEGTCVEWLRRYLQLGN   176
H2-Ld    138   MAAQITRRKWEQAGAAEYYRAYLEGECVEWLHRYLKNGN   176
```

Supplementary Figure 9. Sequence alignment of the α1 and α2 helices of murine MHC-I molecules. Sequence alignment of the α1 and α2 helices of the known surface expressed Qa-2 family (H2-Q7 and H2-Q9), Qa-1^b (H2-Q10), and classical murine MHC-Ia molecules. The consensus sequence is shown above the alignment. Surface-exposed residues, representing potential determinants of receptor specificity, are highlighted in *blue*. Residues whose side-chains contribute to the peptide-binding groove and are not conserved among the murine MHC-I molecules are highlighted in *yellow*, indicating positions likely to contribute to peptide-binding specificity.

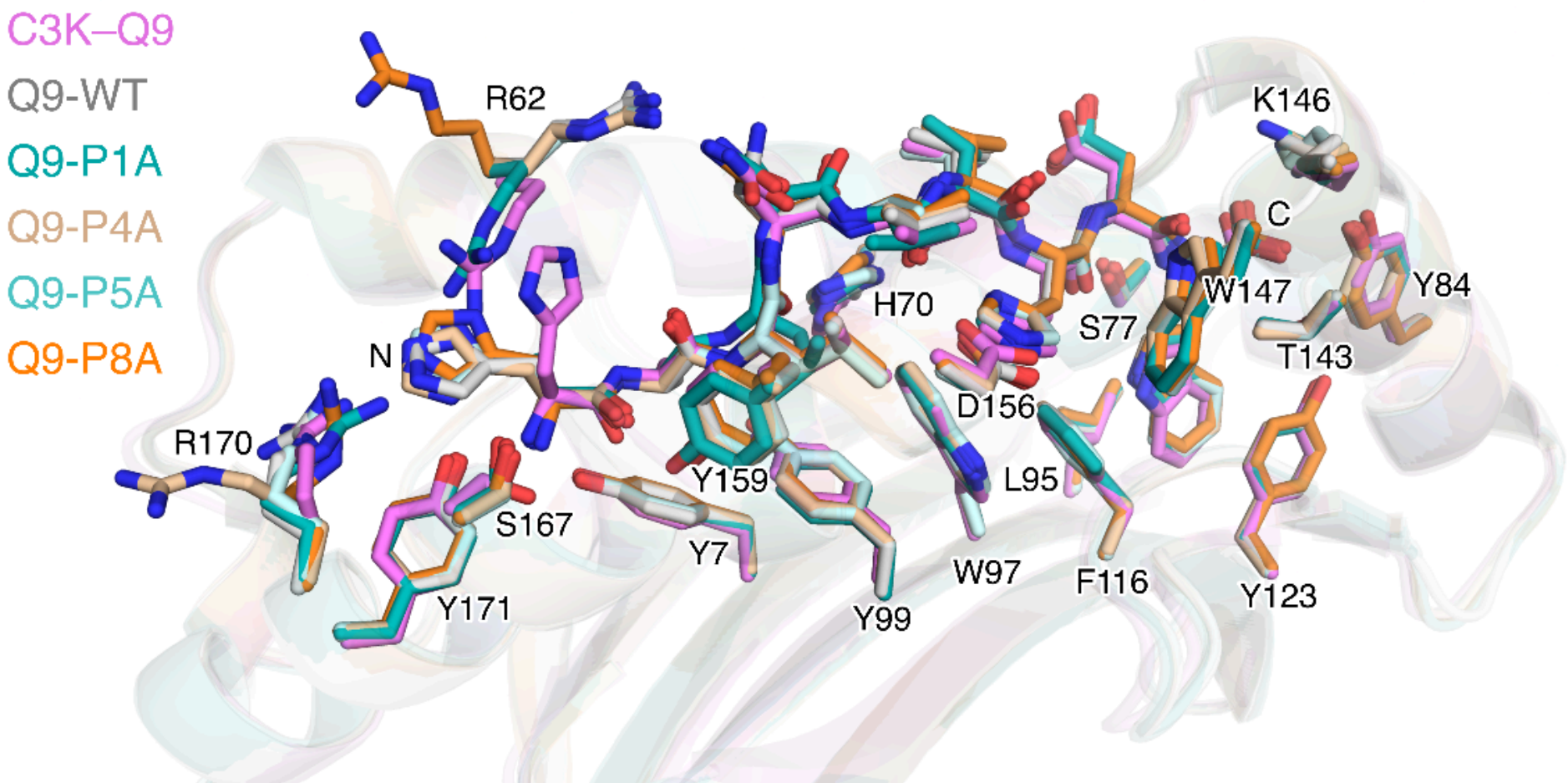

Supplementary Figure 10. Comparison of the Q9 peptide-binding groove among VP2.139 peptide variants. Superposition of the Q9 peptide-binding grooves demonstrates their structural similarity among the VP2.139 peptide variants. The Q9 heavy chain is shown in cartoon, with side-chains of key residues that define the peptide-binding groove shown as sticks. The C3K-bound VP2.139 peptide is coloured *pink*, the wild-type VP2.139 peptide *light grey*, P1A *teal*, P4A *wheat*, P5A *light cyan*, and P8A *orange*.

Supplementary Table 1. Q9-restricted TCR alpha and beta sequences.

Clone	TRAV	TRAJ	CDR3α	TRBV	TRBJ	CDR3β
C3K	TRAV13D-1*04	TRAJ18*01	ASDRGSALGRLH	TRBV2*01	TRBJ1-1*01	ASSQYSTEVF
AH1	TRAV14-2*02	TRAJ40*01	AASEGTGNYKYV	TRBV13-3*01	TRBJ1-1*01	ASRALLDRGANTEVF

Supplementary Table 2. Peptide-binding groove volumes of murine MHC-I molecules.

MHC-I	Peptide-Binding Groove Volume ($Å^3$)
H2-Q9	810.6 ± 50.1
H2-Q10	560.0 ± 40.6
H2-D^b	580.0 ± 50.4
H2-D^d	690.8 ± 40.2
H2-K^b	710.7 ± 20.4
H2-K^d	520.0 ± 30.7
H2-K^k	680.0 ± 20.9
H2-L^d	650.3 ± 80.3

Average peptide-binding groove volumes for the indicated murine MHC-I molecules. Values are reported as mean ± SD. PDBs used for the analysis are as follows: this study and 1FO0, 1G6R, 1K8D, 1KJ2, 1MWA, 1NAM, 1ZT1, 1ZT7, 2FWO, 2OI9, 2OL3, 3ECB, 3PQY, 3TF7, 3TFK, 3TPU, 4MVB, 4MXQ, 4N0C, 4N5E, 5GSV, 5GSR, 5IVX, 5J6G, 5J6H, 5T7G, 5M02, 5WLG, 6G9Q, 6L9L, 7JWJ.

Supplementary Table 3. Calculated docking angles and buried surface areas of published TCR–pMHC-I murine ternaries.

PDB ID	MHC-I	Crossing Angle (°)	Incidence Angle (°)	Tilt Direction	Source	TCR–pMHC BSA (Å²)	TCR–peptide BSA (Å²)	Peptide BSA % of interface
43RH	H2-Q9	48.9	−17.2	N	Viral	2190.2	703.5	32.1
Mean		38.9 ± 11.4	14.7 ± 6.3			1742.4 ± 217.6	602.3 ± 106.5	34.7 ± 5.5
1FO0	H2-K^b	41.2	12.2	C	Endogenous	1405.3	398.0	28.3
1G6R	H2-K^b	22.5	8.5	C	Endogenous	1875.4	543.7	29.0
1KJ2	H2-K^b	30.7	5.9	C	Endogenous	1895.7	508.3	26.8
1MWA	H2-K^{bm3}	22.1	9.0	C	Endogenous	2115.3	665.6	31.5
1NAM	H2-K^b	40.0	11.9	C	Viral	1663.2	443.0	26.6
2OI9	H2-L^d	47.7	8.1	C	Endogenous	1772.4	641.3	36.2
2OL3	H2-K^{bm8}	41.9	13.9	C	Endogenous	1494.4	538.3	36.0
3PQY	H2-D^b	49.8	18.9	C	Viral	1692.0	759.1	44.9
3TF7	H2-L^d	50.1	23.3	C	Endogenous	1424.1	511.9	35.9
3TFK	H2-L^d	45.6	25.8	C	Synthetic	1677.5	615.8	36.7
3TPU	H2-L^d	42.1	11.9	C	Synthetic	2103.1	795.5	37.8
4MVB	H2-L^d	58.4	13.3	C	Endogenous	1748.4	491.8	28.1
4MXQ	H2-L^d	47.4	21.2	C	Endogenous	1596.9	526.8	33.0
4N0C	H2-L^d	45.4	22.1	C	Endogenous	1622.3	640.2	39.5
4N5E	H2-L^d	45.1	22.4	C	Endogenous	1755.3	700.5	39.9
5IVX	H2-D^d	30.1	24.0	C	Viral	2006.2	699.3	34.9
5M02	H2-D^b	41.9	12.8	C	Viral	1702.8	606.7	35.6
5WLG	H2-D^b	18.5	4.9	C	Parasite	2146.5	582.9	27.2
6G9Q	H2-D^b	42.0	13.9	C	Endogenous	1718.3	651.8	37.9
6L9L	H2-L^d	39.1	13.4	C	Synthetic	1705.2	761.4	44.7
7JWJ	H2-D^b	15.3	12.1	C	Viral	1470.7	567.2	38.6

Tilt direction indicates whether the TCR tilts toward the N- or C-terminus of the peptide. Mean = mean ± SD. BSA = buried surface area. $BSA_{TCR–pMHC} = (SASA_{TCR} + SASA_{pMHC}) – SASA_{TCR–pMHC}$. $BSA_{TCR–peptide} = (SASA_{TCR} + SASA_{peptide}) – SASA_{TCR–peptide}$. SASA = solvent accessible surface area.

Supplementary Table 4. C3K–Q9 interactions at the interface.

**Q9 α1-helix**	**TCR contact**	**Interaction type**
Q9-65-Gln	TCRα-94-Ala, TCRβ-55-Met, TCRβ-56-Asp	vdW
Q9-65-Gln $N_{\varepsilon2}$	TCRβ-55-Met O, TCRβ-56-Asp $O_{\delta1}$	H-bond
Q9-66-Ile	TCRα-92-Gly, TCRα-94-Ala	vdW
Q9-69-Gly	TCRβ-55-Met	vdW
Q9-72-Gln	TCRβ-50-Ser, TCRβ-51-Tyr, TCRβ-53-Lys	vdW
Q9-72-Gln $N_{\varepsilon2}$	TCRβ-50-Ser O	H-bond
Q9-72-Gln $O_{\varepsilon1}$	TCRβ-50-Ser O_{γ}	H-bond
**Q9 α2-helix**	**TCR contact**	**Interaction type**
Q9-146-Lys	TCRβ-96-Tyr	vdW
Q9-150-Ala	TCRβ-96-Tyr	vdW
Q9-152-Ile	TCRβ-96-Tyr	vdW
Q9-152-Ile	TCRβ-96-Tyr	π
Q9-154-Glu	TCRα-50-Pro, TCRα-54-Lys	vdW
Q9-154-Glu	TCRα-54-Lys	vdW
Q9-154-Glu $O_{\varepsilon2}$	TCRα-54-Lys N_{ζ}	Salt-bridge
Q9-155-Lys	TCRα-30-Asn, TCRα-31-Ser, TCRα-48-Tyr, TCRα-90-Asp, TCRβ-97-Ser	vdW
Q9-155-Lys N_{ζ}	TCRβ-97-Ser O	H-bond
Q9-158-Ala	TCRα-30-Asn, TCRα-50-Pro, TCRα-65-Ile	vdW
Q9-159-Tyr	TCRα-30-Asn	vdW
Q9-161-Glu	TCRα-65-Ile, TCRα-66-Lys	vdW
Q9-163-Thr	TCRα-28-Ser, TCRα-29-Leu, TCRα-30-Asn	vdW
Q9-163-Thr $O_{\gamma1}$	TCRα-29-Leu O, TCRα-30-Asn $O_{\delta1}$	H-bond
Q9-166-Gln	TCRα-28-Ser, TCRα-68-Arg	vdW
Q9-166-Gln $O_{\varepsilon1}$	TCRα-68-Arg NH1	H-bond

vdW = van der Waals. H-bond = hydrogen bond. Interactions classified for vdW and π as ≤4.0 Å. Interactions classified for H-bonds and salt-bridges as ≤3.5 Å.

Supplementary Table 5. C3K–VP2.139 interactions at the interface.

**Peptide VP2.139**	**TCR contact**	**Interaction type**
P1-His	TCRα-91-Arg, TCRα-92-Gly	vdW
P1-His $N_{\varepsilon2}$	TCRα-92-Gly O	H-bond
P4-Asn	TCRα-30-Asn	vdW
P4-Asn $O_{\delta1}$	TCRα-30-Asn $N_{\delta2}$	H-bond
P4-Asn $N_{\delta2}$	TCRα-90-Asp $O_{\delta2}$, TCRα-93-Ser O	H-bond
P4-Asn	TCRα-90-Asp, TCRα-92-Gly, TCRα-93-Ser, TCRα-96-Gly, TCRβ-97-Ser	vdW
P4-Asn O	TCRβ-97-Ser O_{γ}	H-bond
P5-Val	TCRβ-96-Tyr, TCRβ-97-Ser	vdW
P6-Val	TCRβ-96-Tyr, TCRβ-97-Ser	vdW
P6-Val N	TCRβ-97-Ser O_{γ}	H-bond
P8-Asp $O_{\delta1}$, $O_{\delta2}$	TCRβ-51-Tyr OH	H-bond
P8-Asp	TCRβ-51-Tyr, TCRβ-96-Tyr	vdW

vdW = van der Waals. H-bond = hydrogen bond. Interactions classified for vdW as ≤4.0 Å. Interactions classified for H-bonds as ≤3.5 Å.
